# Optima TB: A tool to help optimally allocate tuberculosis spending

**DOI:** 10.1371/journal.pcbi.1009255

**Published:** 2021-09-27

**Authors:** Lara Goscé, Gerard J. Abou Jaoude, David J. Kedziora, Clemens Benedikt, Azfar Hussain, Sarah Jarvis, Alena Skrahina, Dzmitry Klimuk, Henadz Hurevich, Feng Zhao, Nicole Fraser-Hurt, Nejma Cheikh, Marelize Gorgens, David J. Wilson, Romesh Abeysuriya, Rowan Martin-Hughes, Sherrie L. Kelly, Anna Roberts, Robyn M. Stuart, Tom Palmer, Jasmina Panovska-Griffiths, Cliff C. Kerr, David P. Wilson, Hassan Haghparast-Bidgoli, Jolene Skordis, Ibrahim Abubakar

**Affiliations:** 1 University College London, London, United Kingdom; 2 Burnet Institute, Melbourne, Australia; 3 World Bank, Washington, District of Columbia, United States of America; 4 The Republican Scientific and Practice Centre for Pulmonology and Tuberculosis, Minsk, Belarus; 5 University of Copenhagen, Copenhagen, Denmark; 6 University of Oxford, Oxford, United Kingdom; University of Zurich, SWITZERLAND

## Abstract

Approximately 85% of tuberculosis (TB) related deaths occur in low- and middle-income countries where health resources are scarce. Effective priority setting is required to maximise the impact of limited budgets. The Optima TB tool has been developed to support analytical capacity and inform evidence-based priority setting processes for TB health benefits package design. This paper outlines the Optima TB framework and how it was applied in Belarus, an upper-middle income country in Eastern Europe with a relatively high burden of TB. Optima TB is a population-based disease transmission model, with programmatic cost functions and an optimisation algorithm. Modelled populations include age-differentiated general populations and higher-risk populations such as people living with HIV. Populations and prospective interventions are defined in consultation with local stakeholders. In partnership with the latter, demographic, epidemiological, programmatic, as well as cost and spending data for these populations and interventions are then collated. An optimisation analysis of TB spending was conducted in Belarus, using program objectives and constraints defined in collaboration with local stakeholders, which included experts, decision makers, funders and organisations involved in service delivery, support and technical assistance. These analyses show that it is possible to improve health impact by redistributing current TB spending in Belarus. Specifically, shifting funding from inpatient- to outpatient-focused care models, and from mass screening to active case finding strategies, could reduce TB prevalence and mortality by up to 45% and 50%, respectively, by 2035. In addition, an optimised allocation of TB spending could lead to a reduction in drug-resistant TB infections by 40% over this period. This would support progress towards national TB targets without additional financial resources. The case study in Belarus demonstrates how reallocations of spending across existing and new interventions could have a substantial impact on TB outcomes. This highlights the potential for Optima TB and similar modelling tools to support evidence-based priority setting.

## Author summary

Tuberculosis (TB) remains a leading global cause of death and morbidity, and 85% of deaths occur in countries where resources for TB care and control are limited. Many countries cannot finance all TB interventions or technologies, which means difficult decisions on what to prioritise and publically finance. Modelling tools can help decision-makers set priorities based on evidence, in a systematic and transparent way. This study presents Optima TB, a tool that estimates which allocations of spending across interventions will most likely maximise specified objectives—such as minimising TB deaths, prevalence and incidence. In partnership with local decision-makers and stakeholders, Optima TB was applied in Belarus. Recommendations from the model findings include focussing investment on outpatient rather than inpatient care and actively finding people with TB (e.g. through contact tracing) rather than mass testing of the population. The recommended reallocations of spending could reduce TB prevalence and deaths by up to 45% and 50%, respectively, by 2035 for the same amount of spending. Key stakeholders were engaged throughout the analysis and findings and uncertainty around the results were clearly communicated with decision-makers. The timeliness of the results helped inform national dialogue on TB care reform, among other key policy discussions.

## Introduction

The past decade has seen global improvements in key TB indicators, including incidence and notifications reported by National Tuberculosis Programmes (NTPs). However, while global active TB incidence has decreased at an annual rate of 1.5–1.8%, this fell short of the 4–5% decline required by 2020 to meet the End TB strategy milestones [1, 2]. Furthermore, to meet the End TB 2035 targets of treating at least 90% of all incident cases [3], the rate of active TB notification (estimated at 69% of incidence in 2018) [4] must increase substantially. Diagnosed people must then be linked to effective care and treatment.

Approximately 85% of TB-related deaths occur in low- and middle-income countries, where available resources for TB programmes are scarce [4]. The emergence of new drugs and technologies, such as bedaquiline, GeneXpert tests and geospatial mapping to inform targeted screening, offer additional options in the TB response. However, governments and NTPs are not able to fully finance all available interventions. As such choices must be made regarding which interventions to prioritise and at what level of coverage for populations in need.

Best practice for priority setting involves evidence-based, systematic and transparent decisions that reflect trade-offs, with a high level of stakeholder involvement [5, 6]. When setting priorities, governments of low- and middle-income countries are faced with significant challenges including financial barriers and limited experience with decision-science [6–8]. Modelling tools can support analytical capacity to inform evidence-based decision-making [9, 10]. Allocative-efficiency modelling tools in particular enable the health impact of different interventions or packages of services to be estimated for a given level of spending [10, 11]. In addition, such analyses can support transparent decision making, provided they are carried out with appropriate consultation [10]. Common objectives for TB responses include minimising new TB infections, TB-related deaths, disability-adjusted life years (DALYs), and current and future TB-related costs.

A number of tools currently provide evidence on allocative efficiency for TB responses, including TIME Impact, AuTuMN, SEARO and EMOD [12–16]. These have already been applied in a range of countries and typically simulate (a) TB transmission within and between population groups, (b) TB disease progression, (c) the effects of TB prevention, testing and treatment programmes, and (d) the economic effects of policy choices. However, if developed to enable linking with cost functions, which represent the relationship between intervention spending and corresponding coverage levels, and an optimisation algorithm, mathematical modelling tools can be helpful in determining an optimised resource allocation for defined objectives [17]. Resource optimisation models can also be used to estimate the minimal amount of resources required to achieve specific targets.

This paper presents Optima TB, an open-source tool to support and inform decision-making to improve the TB response. Optima TB is part of the modelling suite of the Optima Consortium for Decision Science (OCDS) [18–20], which has developed and applied disease-specific resource optimisation models in collaboration with governments in over 60 countries, the World Bank, non-governmental organisations, local stakeholders and academic institutions. Optima TB draws on this experience and builds on existing interdisciplinary dialogues between modelers, epidemiologists and government officials. The paper describes how Optima TB was specifically designed to support countries in prioritising available resources for TB control through allocative efficiency analyses. To illustrate the use of Optima TB in country decision-making, a case study in Belarus is presented.

Belarus has the highest proportion of TB drug-resistance worldwide, comprising 38% and 67% of new and retreated cases, respectively. Globally, the median cost of treating drug-resistant TB (DR-TB) is often at least six times higher than treating drug-susceptible TB (DS-TB), and treatment outcomes are less successful (at a rate of 55%) compared with those for DS-TB (82%) [21]. At the time of analysis, the provision of TB treatment in Belarus relied on an ageing infrastructure of costly tertiary care facilities, and ineffective practices such as population-wide mass-screening using chest X-rays [22]. There was thus a need to investigate the cost and impact of the TB response in Belarus, and to revisit the package of services provided by the NTP. These TB care practices and challenges are common elsewhere in Eastern Europe and as such, the findings of this case study will have relevance to other countries [23–27].

The following sections describe how the Optima TB tool was used to determine a recommended package of priority interventions and associated spending allocations, as well as estimate the potential impact on key TB indicators if there were to be a change in policy based on these recommendations.

## Methods

### Optima TB methodology

**Fig 1:**
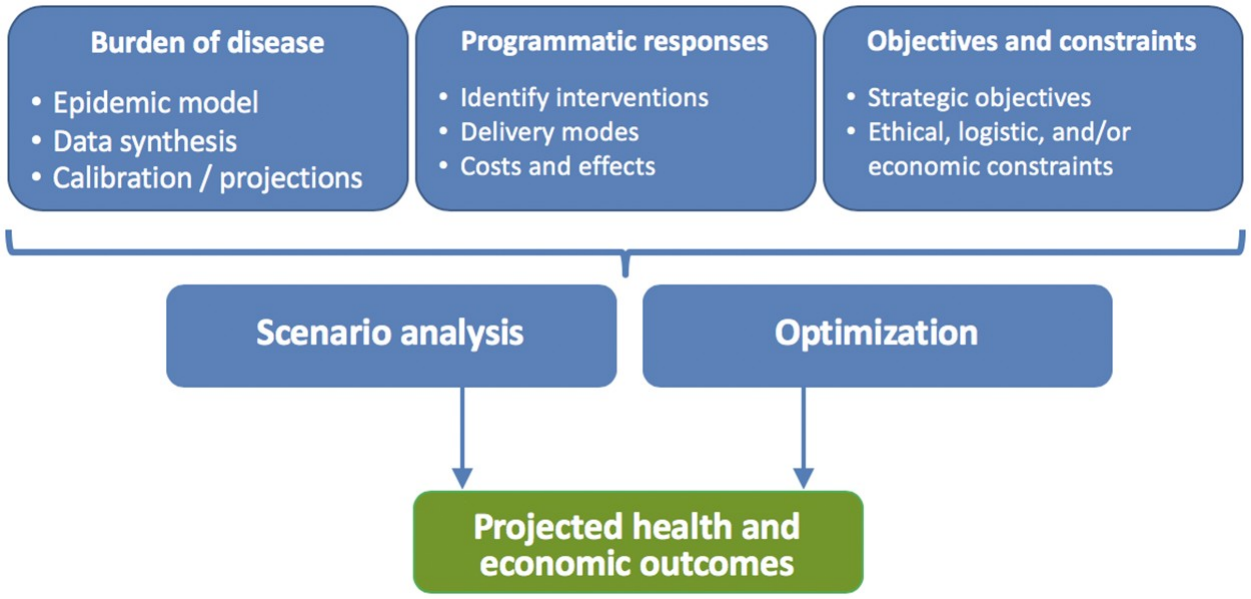
Schematic of an Optima TB analysis (source: OCDS).

The Optima TB incorporates four main components: (a) an underlying epidemiological dynamic disease transmission model to which intervention outcomes are linked; (b) cost-functions that combine data on intervention expenditure and coverage to estimate and project intervention outcomes; (c) objective functions often reflecting national strategic targets, alongside constraints to reflect logistic, ethical, political and financial considerations; and (d) a mathematical optimisation algorithm that combines (a)-(c) to identify the most efficient allocation of resources. These components are depicted schematically in Fig 1.

**Fig 2:**
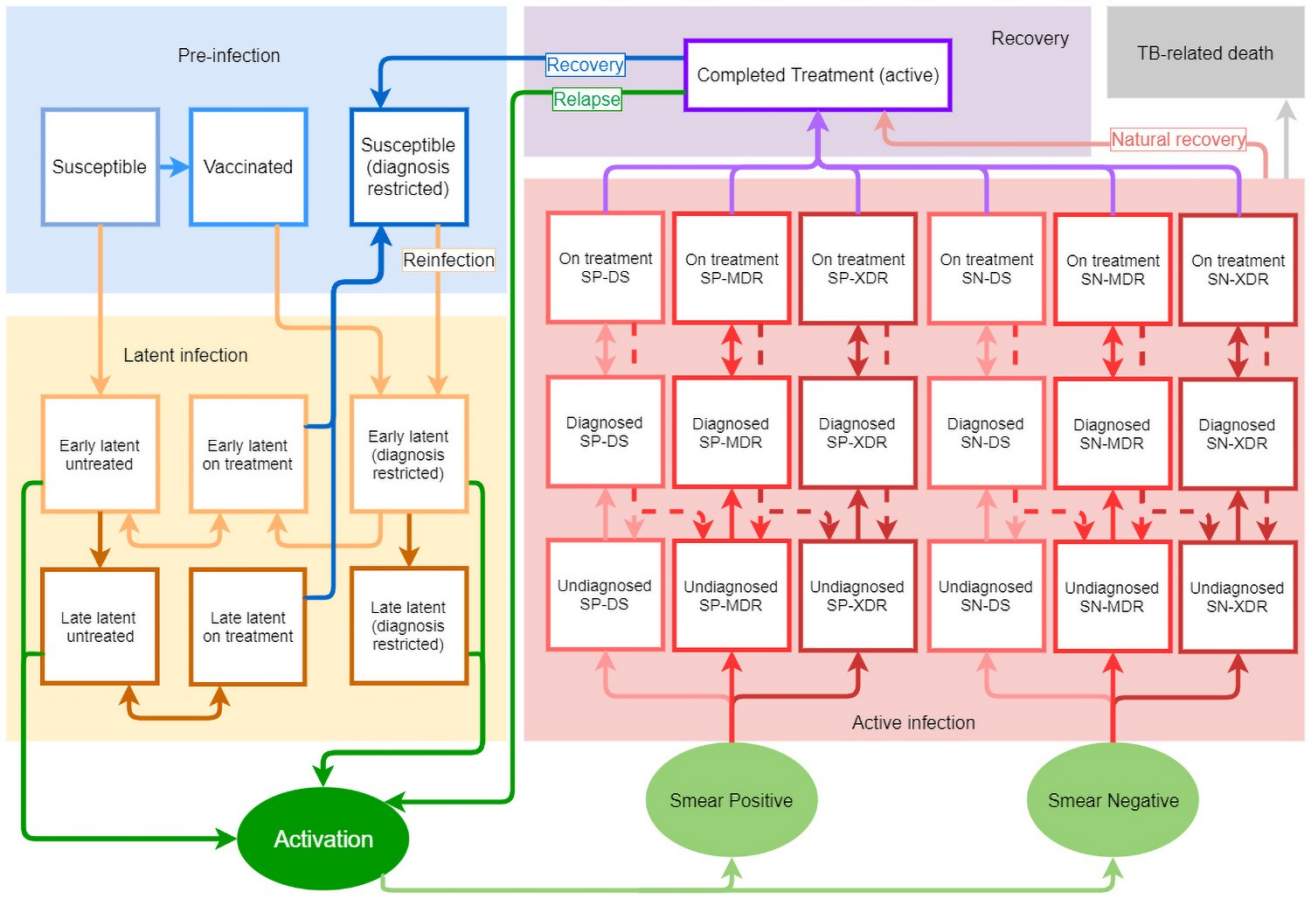
Structure of the epidemiological model at the core of Optima TB. Square boxes in the figure represent compartments, or population sizes, at a given point in time. Ellipses represent junctions, which unlike compartments do not represent population sizes, and only enable the ‘filtering’ of flows of latent activations into either smear-positive or smear-negative, and subsequently into drug-susceptible or a form of drug-resistant TB. Solid lines between boxes and ellipses represent transition rates, or the probability of moving from one compartment to another, within a given timeframe. Dashed lines represent specific transition rates relating to the probability of interrupted treatment or developing multi-drug resistant or extensively drug-resistant TB while on-treatment.

The epidemiological model at the core of the Optima TB tool is represented in Fig 2 and is described below. The mathematical optimisation algorithm used is detailed elsewhere [28]. The version of Optima TB code used for the Belarus analysis is open source and open access (https://github.com/optimamodel/optima-tb). The latest epidemiological model is implemented within the open access Atomica tool (https://github.com/atomicateam/atomica). The user interface and webserver components are written using Sciris (https://github.com/sciris/sciris). The tool is available as part of the “Atomica Applications” suite (https://github.com/sciris/atomica_apps). The interface itself is available at http://tb.ocds.co.

#### Model overview

The model is a compartmental model of disease transmission. While individual based models (IBMs) are best at capturing factors that influence infection on a person-by-person base, this structure was chosen because of the advantages it offers when studying disease transmission in a population on a large scale. Furthermore, although infection progression towards TB disease is a heterogeneous process [29] where the 10% lifetime risk of disease following infection [30] can vary according to age and co-infections [31], Optima TB is able to capture these differences.

Specifically, Optima TB allows for several populations to be defined, each population with its respective structure shown in Fig 2 and each with distinct parameter values. These population structures are then simulated to interact. This includes people with co-morbidities; these populations are modelled with modified parameters for TB disease progression, mortality risk, and co-morbidity treatment coverage such as antiretroviral therapy for people living with HIV.

Accordingly, Optima TB is able to readily capture the following aspects with deterministic modelling:

- Development of TB disease and its severity is age dependent. Children aged under 5 and those infected with latent TB are at higher risk of progressing to active TB [32–34].
- Incidence of TB is higher in individuals with impaired immunity [35]. Consequently, co-morbidities with other illnesses that suppress immunity (e.g. HIV infection, diabetes etc.) often entail a higher probability of developing active TB [36–39].
- TB transmission is much higher in closed and crowded environments such as prisons [40] and mines [41, 42].

#### Equations and parameters

The model structure is depicted in Fig 2 and this section provides mathematical labels for the number of people in each compartment. All individuals are born susceptible, **S**; some of them (usually children) get vaccinated and move into the vaccinated compartment **V**. People who get infected with *Mycobacterium Tuberculosis* first move into the early latent untreated compartment, $L_{e}^{u}$. If they do not develop active TB in the first 5 years after infection, they move into the late latent untreated compartment, $L_{l}^{u}$. The model also considers treatment for latent tuberculosis, represented by the early latent on-treatment and late latent on-treatment compartments, $L_{e}^{t}$ and $L_{l}^{t}$, respectively, along with those successfully treated from latency, *J*. People who received preventive treatment (in the form of vaccination or latency treatment) once reinfected move to their own latency pathway, $L_{e}^{p}$ and $L_{l}^{p}$, where they do not generally have the possibility of getting treated again (except for cases such as people living with HIV (PLHIV) and specific scenarios). This is the ‘diagnosis restricted’ pathway in Fig 2.

If active TB arises, people move into the undiagnosed active disease set of compartments, ***D***^***u***^ (via a single intermediate subclinical compartment, $D_{e}^{u}$; see Eq A in S1 File). There are six undiagnosed compartments; the active TB pathway is divided by smear, SP for positive and SN for negative, as well as by strain type, i.e. drug sensitive (DS), multi drug resistant (MDR) and extensively drug resistant (XDR). A proportion of these individuals get tested and move into a diagnosed compartment set, ***D***^***d***^, similarly divided by SP/SN and DS/MDR/XDR. Those that start treatment move into active-disease on-treatment compartments, subdivided in the same way. Last, individuals who complete active TB treatment move into compartment ***R***, where they remain for two years. If they experience relapse, they move back to compartments ***D***^***u***^, otherwise they move to compartment ***J*** from which reinfection is possible.

The epidemiological motivations behind this modelling design, as well as its equations and parameter definitions, are provided in S1 File, where in-depth discussions about vaccination, latency, reactivation, drug-resistance and TB recurrence can be found.

Naturally, data are not always available to inform the compartment sizes and the transition rates for specific country contexts. Assumptions can be made, but these can arguably lead to more uncertainty and spurious claims than would have arisen if a dynamic feature were entirely excluded. Optima TB was designed from the start to circumvent rigidity; coded in Python and leveraging its object-oriented paradigm, it is easy to include or exclude compartments and transitions as required. For instance, the model uses parameters *δ*_1_ and *δ*_2_ to include the possibility of assuming different fitness for MDR and XDR strains respectively, and also six parameters $\hat{\tau_{i}}$ to study the possible escalation of drug-resistance following incomplete treatment or treatment with non-protocol based regimens. Moreover, the standard form of our TB model treats latency far more comprehensively than is commonly done in other models because of the scarcity of data on latent infections. However, estimates of disease progression from latent to active TB and of untreated active TB outcomes are calculated from very old studies, for which reproducibility would be impossible nowadays, and they are therefore subject to adaptation through calibration (more details are available in S1 File).

Calibration is a multi-step, iterative process. First the model is calibrated against population demographics. Afterwards model estimations are compared with existing data, including estimations of incidence and prevalence from World Health Organisation (WHO) or national data sources [21] disaggregated by sub-populations such as age groups, smear status and/or drug sensitivity.

#### Cost and impact

Optima TB accommodates interventions that directly or indirectly target TB. The former includes prevention, diagnosis and treatment interventions and the latter includes interventions such as behavioural change and awareness campaigns. To include an intervention in an Optima TB analysis, the following must be specified or informed by data: (a) populations served; (b) intervention impact (e.g. diagnostic yield, or probability of successfully completing treatment); (c) unit cost; and (d) intervention coverage. Non-targeted TB programmes, for which a direct impact cannot be assigned, such as management and administration activities, are still costed and included in the analyses but are not included within the optimisation process.

National expenditure on TB is rarely tracked and reported by intervention, but there is often an estimate of total spending on TB and occasionally a disaggregation by broad intervention category such as prevention, diagnosis or treatment. Intervention spending is therefore often estimated using either a top-down approach or a bottom-up calculation, using unit costs and program coverage. By default, the number of people that can be covered by a program can be defined either to scale linearly with expenditure, with a capacity constraint for the maximum number of people that can be covered in a year, or with a saturation value to represent demand constraints such as hard to reach populations. The scaling to reach this saturation value as a percentage of the target population is non-linear, with the marginal unit cost increasing as coverage approaches saturation.

#### Optimisation

Optimisation aims to identify the combination of funded interventions (investments) that achieves the best possible outcome with respect to the optimisation objectives and subject to the optimisation constraints. The objective for optimisation can be to minimize TB-related DALYs, TB-related deaths, total new active TB infections, the prevalence of DS-TB, MDR-TB, or XDR-TB, or any (user-specified) weighted combination thereof. For each intervention, minimum and maximum spending constraints can be specified.

Optimisation can be performed using one of three built-in optimisation algorithms. By default, Optima TB uses Adaptive Stochastic Descent (ASD) [28] implemented by the “Sciris” Python package; this is a gradient-based descent algorithm, which makes stochastic downhill steps in parameter space from an initial starting point, choosing future step sizes and directions based on the outcome of previous steps. Other optimisation algorithms available in Optima TB are particle swarm optimisation [43] via the “Pyswarm” package, which is more computationally expensive but is better able to find global minima if parameter space is complex, and a sequential model-based optimisation algorithm via the “Hyperopt” package [44], which balances the exploration of global and local minima.

### Application in Belarus

This section details the process of applying Optima TB in Belarus, including data collation, model calibration, interventions modelled, and an optimisation analysis of TB spending.

A request for technical support to prioritise TB interventions was made by the Ministry of Health of the Republic Belarus to the World Bank. In addition, an NTP review had found that the Belarus TB response required further alignment with WHO recommendations and guidelines [45]. Following discussions with relevant stakeholders, an allocative efficiency analysis was selected as the preferred approach to inform national priority setting and TB reform processes. A group of key stakeholders was then formed, alongside a smaller working group of local experts from The Republican Research and Practical Centre for Pulmonology and TB. A full list of the stakeholders involved, is included in S2 File. Stakeholders were kept informed throughout the process and reviewed optimisation objectives and results, while the expert working group provided input throughout the analysis, generated and validated assumptions, and reviewed results. Results were presented in a dissemination workshop and an application report was generated and validated by the national team, funders, and stakeholders. The application report for Belarus is posted on the funder’s website (https://openknowledge.worldbank.org/handle/10986/27475) and on the Optima Consortium for Decision Science website (http://ocds.co/tb/applications.html).

#### Data collation, calibration, and validation

Six populations were defined for Belarus as follows: (1) people age 0–4 years, (2) 5–14 years, (3) 15–64 years (without HIV), (4) 65+ years (without HIV), (5) people living with HIV aged 15 years or more, and (6) inmates aged 15 years or more. For Belarus, United Nations (UN) Population Division World Prospects data, local census data and national reports were used to inform population sizes, migration rates, birth rates and non-TB death rates [46–49]. Data on the number of notified TB infections were obtained from the National TB Programme database [50], and other epidemiological data were compiled from local and international publications or reports [51–54]. Key population statistics are provided in Table 1. Intervention cost and expenditure data were sourced from the Belarus TB sub-accounts within the WHO National Health Accounts and other secondary sources [22, 55–57]. Data on the coverage and impact of interventions were sourced from the National TB Programme dataset, and a comprehensive review of local and international literature provided additional data where required [50, 52, 58–71]. Local stakeholders and experts were consulted throughout the analysis to provide input on missing or inconsistent data as well as on epidemic projections.

**Table 1:** Key population statistics.

Population	2005	2010	2015
**Population Sizes**			
0–4	429,281	509,595	577,740
5–14	1,007,770	865,489	921,333
15–64	6,718,570	6,660,400	6,393,500
65+	1,414,080	1,306,960	1,318,510
PLHIV (15+)	15,013	21,040	34,089
Prisoners (15+)	36,948	37,352	33,388
**TB Prevalence**			
0–4	0.03%	0.01%	0.01%
5–14	0.06%	0.06%	0.04%
15–64	0.18%	0.16%	0.15%
65+	0.15%	0.15%	0.14%
PLHIV (15+)	2.34%	2.00%	1.31%
Prisoners (15+)	1.41%	0.70%	0.41%
**TB Incidence**			
0–4	50	24	13
5–14	241	177	138
15–64	3,223	2,980	2,730
65+	594	547	554
PLHIV (15+)	97	110	155
Prisoners (15+)	142	73	42
**TB-related mortality**			
0–4	16	9	4
5–14	62	49	33
15–64	1,131	1,128	884
65+	192	189	165
PLHIV (15+)	59	72	71
Prisoners (15+)	56	29	14

**Fig 3:**
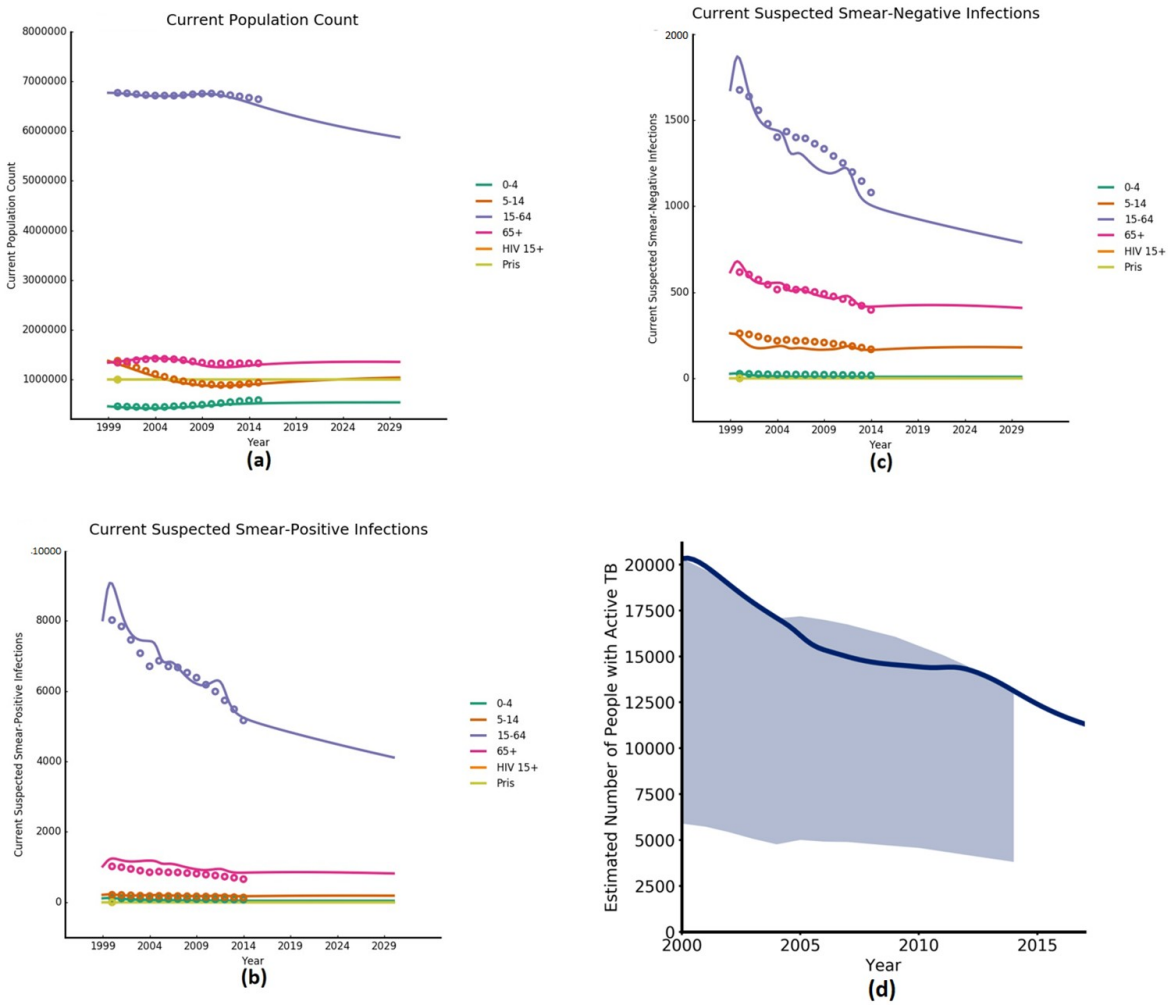
Calibration output graphs. (a) Datapoints are UN Population Division estimates (2000–2015) [49], (b) and (c) datapoints are WHO active TB prevalence upper-bound estimates disaggregated by population group (2000–2014) [53] and (d) shaded area represents confidence intervals of WHO active TB prevalence estimates for total population (2000–2014) [53].

Subsequently, the model was calibrated through manual fitting to match closely to the annual numbers of notified TB infections, as well as estimates of key TB indicators such as active TB incidence/prevalence (Fig 3) and latent TB prevalence. Parameters with the greatest uncertainty were selected for adjustment during the calibration process. These were the: (1) transmission rate, (2) probability of progressing from early- or late-latent TB to active TB, (3) re-infection rate of people recovered from active TB, and (4) the proportion of early- versus late-latent TB infection. Model inputs, calibration parameter values, and epidemic projections were compared against peer-reviewed publications, parameter values used in other modelling studies, and secondary data and estimates and epidemic projections as part of the calibration process. Further details on the process as well as the estimates used, are contained in the published report [72].

#### Interventions modelled

Different types of interventions can be included in Optima TB, such as preventative therapy, screening and diagnosis, active TB treatment, and other TB-related activities such as management, procurement, or human resources. Table 2 details the interventions modelled in the Belarus analysis. To derive the unit costs for each intervention listed in Table 2, a mixture of top-down and bottom-up costing was undertaken using a calculated average cost per diem of inpatient or outpatient visits (see S2 File). National drug procurement records for 2016 and other secondary data were consulted, as well as expert opinion in cases such as mass-screening [22, 55–57]. Coverage and impact data were then used to generate a cost-function for each intervention, with the exception of non-targeted interventions not considered in the optimisation analysis such as management or procurement activities. Interventions that were not targeted in the optimisation analysis either do not have a direct measurable impact on the epidemic (non-targeted interventions), such as procurement activities, or did not have sufficient data at the time as was the case for alcohol interventions and palliative care.

**Table 2:** Interventions included in the Belarus analysis.

Intervention	Population(s) targeted	Description	Unit cost* (US$, 2015)	Spending (US$, 2015)
**Screening and diagnosis interventions**				
Active Case Finding	15–64; 65+; PLHIV	Screening of high-risk groups that are considered at high risk of developing active TB (e.g. PLHIV, homeless, people who inject drugs etc.) with chest X-ray or fluorography	$1.00	$942,159
Incentivised Active Case Finding	15–64; 65+; PLHIV	Active case finding with subsided transportation for healthcare workers and incentives for each case identified	$5.20	Prospective intervention
Contact Tracing	All populations (contacts of people with active TB)	Tracing of household contacts of people diagnosed with active TB, and screening using symptomatic questionnaire, smear-sputum microscopy, and GeneXpert	$1.00	$7,046
Incentivised Contactì Tracing	All populations (contacts of people with active TB)	Contact tracing with subsided transportation for healthcare workers and incentives for each case identified	$5.20	Prospective intervention
Symptomatic Diagnosis (including Xpert)	All populations	screen questions followed by smear-sputum microscopy, Gene Xpert and liquid culture for patients that present at a health facility with symptoms of TB	$39.98	$770,489
Mass-Screening	All populations	Yearly general population-wide mass-screening using chest X-rays and fluorography	$1.00	$9,758,596
**Treatment for active TB**				
Hospital Focused DS-TB	People receiving treatment for DS-TB	Treatment for DS-TB with hospitalisation for 60 days out of a total treatment duration of 180 days	$2,609.72	$7,283,723
Hospital Focused MDR-TB	People receiving treatment for MDR-TB	Standardised treatment regimen for multidrug-resistant TB (MDR-TB), with hospitalisation for 180 days out of a total treatment duration of 540 days	$14,158.05	$12,884,772
Hospital Focused XDR-TB	People receiving treatment for XDR-TB	Treatment for extensively drug-resistant TB (XDR-TB) with available second and third-line drugs, with hospitalisation for 240 days out of a total treatment duration of 660 days	$20,483.19	$7,445,214
Ambulatory DS-TB	People receiving treatment for DS-TB	WHO recommended outpatient service delivery, with hospitalisation only during the intensive phase of a given regimen or until smear conversion. Involves hospitalisation for 14 days out of a total treatment duration of 180 days	$1,877.83	Prospective intervention
Ambulatory MDR-TB	People receiving treatment for MDR-TB	WHO recommended outpatient service delivery, with hospitalisation only during the intensive phase of a given regimen or until smear conversion. Standardised MDR-TB regimen with hospitalisation for 45 days out of a total treatment duration of 540 days	$10,196.29	Prospective intervention
Ambulatory MDR-TB Short-Course	People receiving treatment for MDR-TB	Short-course MDR-TB regimen. Involves hospitalisation for 30 days, out of a total treatment duration of 315 days	$4,520.46	Prospective intervention
Ambulatory XDR-TB	People receiving treatment for XDR-TB	WHO recommended outpatient service delivery, with hospitalisation only during the intensive phase of a given regimen or until smear conversion. Treatment for XDR-TB with available second and third-line drugs, with hospitalisation for 60 days out of a total treatment duration of 660 days	$15,440.95	Prospective intervention
Incentivised Ambulatory DS-TB	People receiving treatment for DS-TB	Similar to the ambulatory DS-TB intervention, but incorporates financial incentives (food packages, outcome-based financing) based on the Mogilev District pilot project	$2,215.36	Prospective intervention
Incentivised Ambulatory MDR-TB	People receiving treatment for MDR-TB	Similar to the ambulatory MDR-TB intervention, but incorporates financial incentives (food packages, outcome-based financing) based on the Mogilev District pilot project	$11,324.79	Prospective intervention
Incentivised Ambulatory MDR-TB Short-Course	People receiving treatment for MDR-TB	Similar to the ambulatory MDR-TB short-course intervention, but incorporates financial incentives (food packages, outcome-based financing) based on the Mogilev District pilot project	$5,099.96	Prospective intervention
Incentivised Ambulatory New Drugs MDR-TB	People receiving treatment for MDR-TB	Similar to the ambulatory MDR-TB intervention but with new and repurposed drugs, including bedaquiline and linezolid, added to the background regimen. Incorporates financial incentives (food packages, outcome-based financing) based on the Mogilev District pilot project	$14,797.00	Prospective intervention
Incentivised Ambulatory XDR-TB	People receiving treatment for XDR-TB	Similar to the ambulatory XDR-TB intervention, but incorporates financial incentives (food packages, outcome-based financing) based on the Mogilev District pilot project	$16,782.95	Prospective intervention
Incentivised Ambulatory New Drugs XDR-TB	People receiving treatment for XDR-TB	Similar to the ambulatory XDR-TB intervention but with new and repurposed drugs, including bedaquiline and linezolid, added to the background regimen. Incorporates financial incentives (food packages, outcome-based financing) based on the Mogilev District pilot project	$23,036.00	Prospective intervention
Involuntary Isolation MDR-TB	People receiving treatment for MDR-TB	Treatment for people with MDR-TB with a history of adherence problems in a dedicated facility monitored by police	$45,588.00	$10,895,532
Involuntary Isolation XDR-TB	People receiving treatment for XDR-TB	Treatment for people with XDR-TB with a history of adherence problems in a dedicated facility monitored by police	$45,588.00	$5,698,500
**Preventative interventions**				
IPT for General Population	All populations (contacts of people with active TB)	Treatment of latent TB Infections with 6-months Isoniazid therapy in general population TB-contacts	$11.52	$10,575
IPT for PLHIV	PLHIV	Treatment of latent TB Infections with 6-months Isoniazid in PLHIV	$11.52	$2,477
BCG Vaccination	0–4 years	Bacillus Calmette–Guérin (BCG) vaccination for newborns	$1.32	$528,000
**Interventions or activities not included in the optimisation analysis (spending fixed)**				
Solid Culture	People with MDR- or XDR-TB	Cost of solid culture testing to identify and confirm resistance types of MDR-TB and XDR-TB	$1.43	$362,108
Line Probe Assay	People with MDR- or XDR-TB	Cost of line probe assay (LPA) testing to identify and confirm resistance types of MDR-TB and XDR-TB	$16.16	$78,938
Tuberculin Skin Test	0–4 years; PLHIV; contacts of people with active TB	Cost of conducting a tuberculin skin test (TST) test to diagnose latent TB infections	$4.32	$475,200
Palliative Care MDR/XDR	People receiving treatment for MDR- and XDR-TB	Palliative care for MDR-TB and XDR-TB patients with a history of non-adherence, repeated treatment failure, and adverse reactions	$5,108.00	$2,894,426
Alcohol Intervention	People receiving treatment for active TB with problems of alcohol abuse	Cost of alcohol programmes to support adherence to TB treatment regimens	n/a	$210,000
Management (Including HR)	n/a	Administrative costs	n/a	$892,061
Procurement Costs	n/a	As per TB sub-accounts of NHA	n/a	$649,349
Other Costs	n/a	As per TB sub-accounts of NHA	n/a	$255,055

* Annualised treatment costs are used in the tool.

Note: All unit costs include the cost of service delivery in addition to medicine, test kits, etc. All treatment unit costs are per DS-TB, MDR-TB or XDR-TB case.

#### Optimisation analysis

After extensive consultation with a TB working group of stakeholders in Belarus, three key output indicators were identified for the optimisation analysis: (1) TB-related deaths, (2) all TB infections (i.e. prevalence), and (3) new TB infections (i.e. incidence). To determine the optimised allocation of resources, the working group agreed to define an objective function for the ASD model algorithm to minimise these three key output indicators, given the local epidemic parameters and data, cost of delivering services, and subject to defined constraints. Before running the optimisation analysis, constraints shown in Table 3 were therefore defined by key stakeholders and local experts to reflect logistic, ethical, political, and financial barriers for scaling-up or defunding specific interventions. Additionally, time-dependent constraints were included to reflect realistic timings for changes in the implementation of interventions. Changes in intervention spending between 2015 and target spending levels were capped either at a maximum change of 30% per year for existing interventions, or at a maximum of US$1M for new interventions for the first year and 30% in subsequent years, until the optimised level of spending on an intervention is reached.

**Table 3:** Interventions with defined constraints in the optimisation analysis.

Intervention name	Minimum budget constraint relative to 2015 intervention spending	Maximum budget constraint relative to 2015 intervention spending
BCG vaccination	100%	100%
Testing: TST^1^, LPA^2^, and solid culture testing	100%	100%
Mass-screening (including X-ray)	50%	-
Active case finding for key populations	100%	-
Hospital-based treatments for DS-, MDR- and XDR-TB	30%	-
Palliative care	40%	40%
Involuntary isolation for MDR- and XDR-TB	20%	-

^1^ Tuberculin Skin Test: TST.

^2^ Line Probe Assay: LPA.

Interventions for which data on impact were not available, in the form of yields and sensitivities for screening or diagnosis and relative risks for treatment outcomes, were treated as fixed costs and excluded from the optimisation analysis. To achieve the optimisation objective under set constraints, the optimisation analysis aimed to address the following two questions:

1. What is the optimised allocation of TB spending and associated programme coverage levels, to minimise TB mortality, prevalence, and incidence in Belarus between 2017 and 2035?
2. How much progress will be made toward national and international targets under optimised resource allocations in Belarus compared with continuation of the 2015 response?

## Results

### Optimised TB spending allocation in Belarus

**Fig 4:**
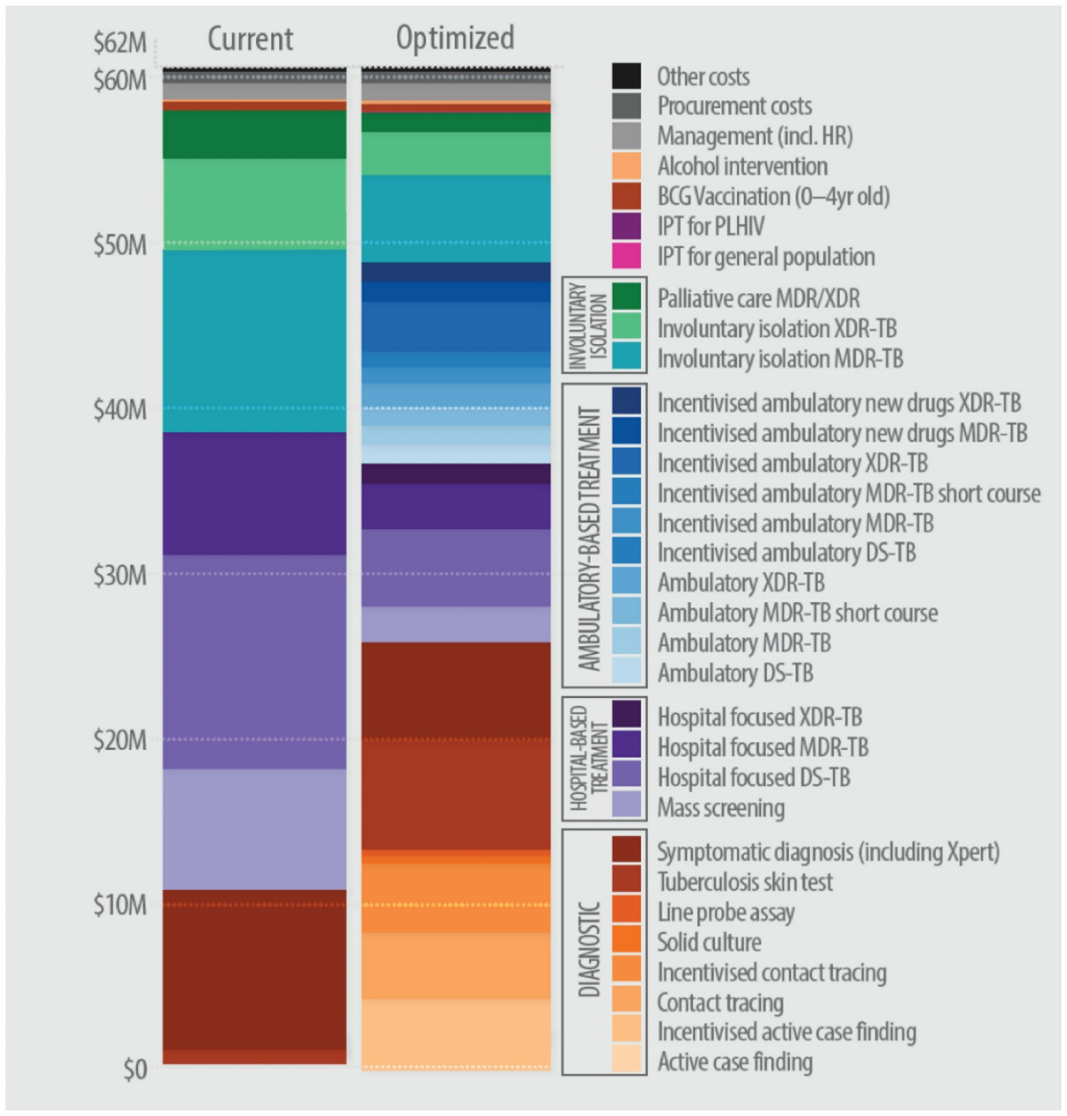
National TB spending in Belarus in 2015 compared with optimised annual budget to minimise active TB prevalence and incidence and TB-related mortality by 2035.

In 2015, an estimated US$61.8 million was spent on the TB programme and TB-related activities in Belarus. The distribution of total TB spending across the various TB interventions in 2015 is shown in Fig 4. Annual population-wide mass-screening with chest X-rays accounted for one-sixth of total TB spending (US$9.8 million), with little investment in contact-tracing and other targeted active case finding interventions. Approximately 45% of total TB spending was invested in hospital-focused interventions (US$27.6 million), of which 74% was on DR-TB treatment (US$20.3 million). Another significant portion of total TB spending (approximately 25%) was on involuntary isolation facilities for drug-resistant treatment of patients at high-risk of loss-to-follow up, with a unit cost of US$45,588.

The mathematically optimised allocation of TB spending that would simultaneously minimise active TB incidence and prevalence and TB-related mortality in Belarus according to our model is shown in Fig 2. Overall, recommendations from the analyses are that spending be shifted from hospital-focused interventions to outpatient treatment. Similarly, investment in annual mass-screening should be de-prioritised in order to prioritise targeted active case finding strategies. Spending on hospital focused treatment and involuntary isolation is reduced by 60% in the optimised budget compared with the 2015 spending allocation. In turn, spending on more cost-effective outpatient treatment interventions is increased to 40% of the optimised spending on treatment, equivalent to 20% of the total targeted TB budget. These reallocations could save around 30% of total treatment expenditure for re-investment in other interventions, while treatment coverage for people diagnosed with TB would increase from 81% to 90%. Savings from the use of more cost effective outpatient treatment and reduced spending on mass screening, should ideally be reinvested to expand targeted screening and diagnosis. Rapid diagnostic testing, targeted active case finding, and contact-tracing interventions are recommended for prioritisation, comprising 30% of total TB spending or 80% of spending on screening and diagnosis.

### Projected impact of optimised spending on the TB epidemic in Belarus

**Fig 5:**
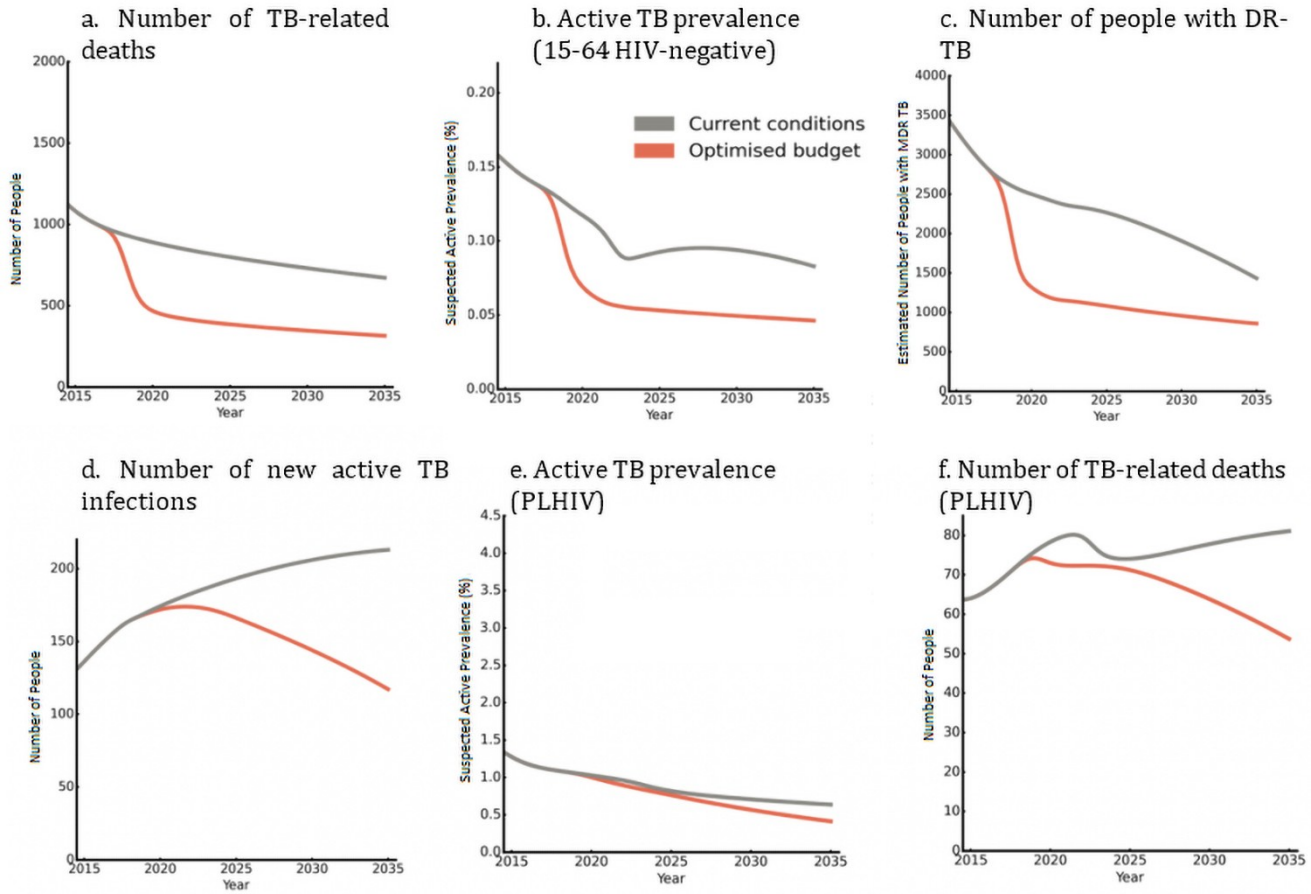
Projected impact of 2015 and optimised allocations of national TB spending on key TB indicators among HIV-negative adults and people living with HIV from 2015 to 2035.

Epidemiological projections are shown in Fig 5, where the grey lines assume that the amount and allocation of TB spending across interventions in 2015 is maintained until 2035. The prevalence of TB is projected to decrease rapidly among HIV-negative populations aged 15–64 years, before stabilising around 2023. TB-related deaths are also estimated to decrease, although steadily. While there is a projected gradual decrease in the prevalence of TB among people living with HIV, the number of new TB infections and TB-related deaths are projected to increase over this period—driven by a projected increase in the number of people living with HIV. In line with the projected overall decrease in TB infections, the number of people with DR-TB is estimated to decrease rapidly, reducing by 50% by 2035 compared with 2015 levels.

An optimised allocation of national TB spending could yield significant improvements in key TB indicators, as shown in Fig 5. Among HIV-negative populations, the model estimates that an optimised allocation of spending could lead to a 45% reduction in adult TB prevalence by 2035 compared with the existing allocation. Similarly, an optimised allocation of spending is estimated to reduce TB-related deaths by 50% by 2035 compared with non-optimised spending allocations, and by 70% compared with 2015 levels. In addition, an estimated 40% of all DR-TB infections in Belarus can be averted by 2035 through optimised reallocations in spending. Among people living with HIV, between a 30% and 45% reduction in new infections, prevalence, and mortality could be realised by 2035, though the number people living with HIV with active TB is very small.

### Progress toward national and international TB targets

**Fig 6:**
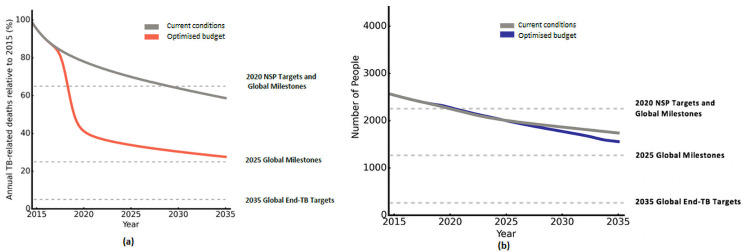
Progress toward national and global TB targets for (a) TB mortality and (b) new TB infections in the 15–64 age group, under 2015 and optimised allocations of national TB spending.

The estimated progress of 2015 and optimised allocations of spending toward national and international targets is shown in Fig 6. The year 2015 is considered as the baseline at 100%, while different targets and milestones are indicated by the dashed lines. Under 2015 spending allocations, it is estimated that no milestones or targets will be met aside from the 2020 National Strategic Plan (NSP) target for incidence. The national NSP target of a 35% reduction in TB-related deaths by 2020 will likely be missed, with the necessary reduction achieved only by 2030. If programmatic spending is reprioritised optimally, projections suggest that 2020 NSP targets for both incidence and TB-related deaths could be met. While neither global milestones nor targets are met under an optimised combination of interventions with 2015 spending levels, more significant progress will likely be made towards mortality targets relative to incidence.

## Discussion

Optima TB is an open-access tool for allocative efficiency modelling, designed to inform priority setting for TB responses. Grounded in available data, it enables different populations and co-morbidities to be defined to capture epidemic heterogeneities. This paper illustrates the potential of Optima TB analysis findings to inform NTP discussions on priority setting and TB response reform via a case study in Belarus. An optimised allocation of TB spending in Belarus was estimated to reduce TB prevalence and mortality by up to 50%, compared with prior programme approaches. The main factors behind the gains are shifts from annual population-wide mass-screening and inpatient focused care, to active case finding strategies and outpatient focused care. Savings from these reallocations could be reinvested in screening and diagnosis, prioritising higher-yield interventions needed in Belarus such as contact tracing and rapid diagnostic testing [50, 59, 61, 73]. In addition, transitioning to cheaper outpatient care could reduce overall treatment spending by 30% for reallocations to other intervention, with no estimated reduction in the number of people on treatment. The average unit cost of an inpatient day is three or four times more than that of an ambulatory day for DS-TB or DR-TB care, respectively (see Table A in S2 File), driven by greater staff, overhead and capital costs. Shifts in spending from inpatient to outpatient focused care are therefore recommended, in line with findings from local literature and WHO guidelines [22, 74–76], which would offset reductions in total treatment spending and enable the NTP to cover approximately 90% of all people diagnosed with active TB. As well as reductions in prevalence and mortality, scaling up care for people with active TB through these reallocations may result in modest gains in incidence over time, as overall levels of infectiousness are reduced through increased diagnosis and treatment in the model.

A central focus of the Belarus NTP is to provide appropriate care for people with DR-TB and to address the significant burden of drug-resistance in the country. Existing practices involve lengthy inpatient stays of 180 days (six months) on average before discharge following smear conversion. An optimised shift in spending from inpatient-focused care to outpatient DR-TB treatment, and to drug-regimens comprised of new and repurposed drugs such as bedaquiline and linezolid, is estimated to reduce the number of DR-TB infections by around 40%. Reduced prioritisation of inpatient care would enable the NTP to provide DR-TB services to a greater number of those in need, and free up funds that can be invested in newer drug regimens with proven efficacy [77]. Furthermore, it is important to note that the estimated gains in this analysis from prioritising outpatient care do not include considerations from the patient perspective. Many studies have highlighted the substantial indirect costs and loss of earnings experienced by patients due to hospitalisation [78, 79].

While it is estimated that 2020 NSP targets for incidence and mortality could be met under optimised allocations of spending,progress would still fall significantly short of meeting the global 2025 TB milestones and 2035 End TB Strategy targets. Other modelling studies have also estimated that countries may fall short of global milestones and targets [80]. To meet global targets in Belarus, as in other countries, new technologies and interventions will likely be required as well as substantial increases in cost-effective investment. Overall, the TB response in Belarus is largely focused on curative rather than preventative interventions (Table 2). Only the latter can reduce vulnerability to TB and the rate at which the large pool of people with latent TB progress to active TB each year through the pathways shown in Fig 2 [81, 82]. The insufficient progress towards global targets for reductions in TB incidence estimated in this analysis supports a large body of existing literature advocating for a more holistic response to the global burden of TB, which is based on an understanding that social determinants of disease such as living conditions and nutritional status significantly impact disease progression to active TB [81, 83–87]. A recent modelling study suggests that the expansion of social protection programmes would yield significant accelerated progress toward global TB targets [88].

Overall, baseline projections in this analysis based on 2015 TB spending allocations appear to be largely in line with recent estimates of key TB indicators for Belarus. For example, the 3400 incident active cases projected in this analysis for 2018 (see Figs A-C in S1 File) falls within the active TB incidence estimates by the WHO for 2018 (between 2300 and 3700) [89]. However, while modelled annual TB-mortality at the time of analysis (1200) for 2014 was higher than the upper-bound of WHO estimates (870), [90] the latter have recently been revised and reduced by approximately a third for 2014 (580) [91]. Mortality projections from the analysis are therefore no longer in line with recent estimates.

In addition, while this analysis was carried out before the publication of guidance for country-level TB modelling [92], the analysis followed the principles listed in the guidance document (see S3 File for details). The timeliness of the Belarus analysis helped inform dialogue on national TB care, including a round-table consultation organised by the WHO country office in Minsk in 2017 and a regional reform meeting in Bishkek, during which cornerstones of reform were agreed [93]. This analysis has informed ongoing activities for TB financing and planning by the WHO Regional Office for Europe and Ministry of Health [94], and the findings have helped advocate for more and better quality ambulatory care. Recently, a national primary healthcare workers scheme has been introduced, which involves bonus payments for the provision of TB services (such as bonus payments of US $5 per day for visits made to TB patients’ homes by nurses for treatment observations [95]). That said, impact assessments are required to establish whether this Optima TB application influenced actual reallocations in spending and improved TB control because of any changes in decision making.

### Strengths and limitations

A number of TB epidemiological modelling tools exist to support country-level decision-making, including TIME Impact, AuTuMN, SEARO, VI, and EMOD among others [12–14, 16, 96]. While most comprehensive models nowadays include the study of MDR-TB cases [12–14, 16, 96], only a few have an explicit structure able to capture co-morbidities or high-risk groups [12, 14, 96] and, of them, only Optima TB and AuTuMN [12] explicitly analyse XDR cases. More specifically, TIME and AuTuMN enable users to run a variety of epidemic scenarios, and TIME Impact can be combined with the OneHealth Tool to produce detailed costings of TB interventions and guide NTP implementation by assessing health system components such as infrastructure or human resource needs. In addition, linking to OneHealth allows for private sector considerations to be incorporated within a TIME model analysis, which is key in certain contexts, such as in India [97]. However, efforts to develop tools that can estimate a mathematically optimised allocation of spending across a number of interventions to maximise a set of desired objectives have only recently begun [12]. The Optima TB tool draws on existing epidemiological tools and research in allocative efficiency. Other Optima tools have been applied in over 60 countries, and follow-up impact assessments have shown that Optima HIV studies have influenced actual allocations in HIV spending in some countries such as Sudan and led to improvements in disease control [10, 11, 98–100].

There are several additional characteristics particular to the Optima TB tool. First, the explicit care-cascade structure allows for the model to be directly initialised and parametrised using country-provided data such as notified cases, number of people initiated on treatment, and TB-related deaths without solely relying on incidence estimations. This means that the modelling output can more accurately represent the setting specific TB epidemic. Another novel aspect that, to our knowledge, is unique in comparison to other TB tools, is a secondary latency pathway. This has multiple advantages as it not only allows individuals who had a previous history of infection to be distinguished, it also allows to study different preventive strategies that confer partial protection against reactivation and disaggregate these individuals from the general population. Currently, the implementation of preventive strategies such as treatment of LTBI, is generally limited to only direct contacts of active cases and HIV positive individuals, the model structure includes detailed capture of TB latency allowing for the testing of new strategies and their impact on the entire population. Key populations can also be flexibly defined and targeted in Optima TB.

One of the strongest design goals of Optima TB has been to ensure that all available epidemic data and estimates for TB for a given setting can be fully utilised by the model, while also maintaining an accessible interface that requires a minimum of data input. Ongoing work will continue to focus on updating default global values for model parameters to represent TB transmission, progression, and treatment efficacy, to incorporate future TB research findings and WHO recommendations. Future model development is ongoing and may also include an additional pre-reactivation compartment for latent TB if a setting is using or plans to implement new diagnostic techniques capable of identifying people at higher risk of latent TB reactivation with a high degree of sensitivity [101].

As with any deterministic model, there are limitations to our approach. These are mainly related to the limited individual-level detail typical of population-level models and the homogeneity between individuals belonging to the same sub-population. Moreover, as is the case with any modelling framework, the quality of the modelling output is tightly connected to the amount and quality of data informing it, particularly so here because of the use of country-informed data. Also, calibration plays a strong role in obviating the paucity of information on epidemic parameters related to infection progression.

Several key limitations to the case study must be considered when interpreting the results. First, model projections are only as reliable as the data that informs them. For Belarus, as for other countries, there are gaps, errors and inconsistencies in and between different datasets, with diminishing quality when data are disaggregated by sub-population. Commonly missing values for TB prevalence or intervention spending means that these values must be estimated, and assumptions have to be made, as informed by local experts. In addition, determining a mathematically optimised allocation of spending is dependent on the availability of estimates on the effectiveness of individual interventions. Optima TB aims to inform resource allocation across interventions based on the defined cost-coverage relationship for a given intervention. If a given intervention can be delivered more efficiently for example through lower costs for the same treatment regimen, this would affect the underlying ranking of the cost-effectiveness of the interventions and thus resource allocation decisions. Although some effectiveness parameters were informed by national data, others were sourced through a review of global literature. Effectiveness can vary across different countries and settings and the latter, therefore, may not be contextually representative. Due to a lack of data, TB interventions for inmates are not included in estimates of national TB spending or within the optimisation analysis.

At the time of this analysis, the capacity for comprehensive uncertainty analyses was not included in the Optima TB model. The updated Optima TB model allows uncertainty intervals to be specified for every input parameter, including key calibration parameters such as transmission and progression rates for a given setting in addition to epidemiological inputs. Uncertainty in model outputs is then generated by sampling from the distributions for each input parameter, resulting in a distribution of values for each output. In its most basic form, these can be used to produce confidence intervals for each output, but the full distributions allow covariance and higher order statistics to be analysed as well.

In addition, our assumption about the nature of the relationship between intervention spending and coverage in Belarus did not consider the possibility of diminishing marginal returns. Last, projections generated by the model use parameter values from the most recent year for which data are available. Intervention impacts were assumed not to change with time and simulations also did not assess varying allocations of resources over time. Despite this, the optimised allocation is based on the time horizon from 2017 to 2035 in order to ensure that immediate gains do not come at the expense of adverse impacts later on and to gauge progress toward international targets with optimised allocations. In practice, however, optimised allocations should be revisited for timelines longer than three to five years.

## Conclusions

The Optima TB tool was applied in Belarus to determine an optimised allocation of national TB spending across TB interventions to best address defined objectives. A shift in programmatic priorities could reduce TB prevalence and mortality by 50% by 2035, compared with a continuation of the 2015 response. Similar reductions of around 40% in the number of people with drug-resistant TB could be achieved, which is a key priority of the national TB programme. These gains would be achieved through shifts from annual population-wide mass-screening and inpatient focused care, to active case finding strategies and outpatient focused care. Optima TB applications aim to use existing country data to help initiate or support dialogue between national TB programme managers and Ministries of Health and Finance, to generate evidence-based findings to inform decisions and to reduce the burden of TB. Follow-up impact assessments will be required to determine whether this Optima TB application influenced future TB resource reallocations in Belarus, and whether there is evidence of improved TB control because of any changes in decision making.

## Supporting information

S1 File Transmission model and epidemiological rationale. Equations and table of parameters (Table A in S1 File) are included, as well as epidemiology background justifying the model’s structure. Additional results are also included: (Fig A in S1 File) projections of new TB infections to 2035; (Fig B in S1 File) number of active TB infections with and without funding to current TB programs; and (Fig C in S1 File) projections f yearly TB deaths to 2035.
(PDF)

S2 File Intervention cost and effect inputs, including. (Table A in S2 File) TB treatment and care cost assumptions; (Table B in S2 File) TB treatment intervention effectiveness inputs; and (Table C in S2 File) TB screening and diagnosis intervention inputs for yield.
(DOC)

S3 File Summary of modelling process in relation to TB MAC country level modelling guidance. (DOCX)
