# Supplementary material for: Optima TB: A tool to help optimally allocate tuberculosis spending
Source: PLoS Comput Biol. 2021 Sep 27;17(9):e1009255. doi: 10.1371/journal.pcbi.1009255 (PMC8496838; doi:10.1371/journal.pcbi.1009255)
Supplement: S1 File — Equations and table of parameters (Table A in S1 File) are included, as well as epidemiology background justifying the model’s structure. Additional results are also included: (Fig A in S1 File) projections of new TB infections to 2035; (Fig B in S1 File) number of active TB infections with and without funding to current TB programs; and (Fig C in S1 File) projections f yearly TB deaths to 2035. (PDF) [file pcbi.1009255.s001.pdf]

Supplement 1: Transmission model and epidemiological rationale

The Model

The evolution of a population, distributed across compartments in Fig 2 in the main document, can be written in equation format as

$$\begin{aligned}
\dot{S} =&\ b-(\nu+D_1+\gamma D_2+\mu)S\\
\dot{V} =&\ \nu S-[\xi_1(D_1+\gamma D_2)+\mu]V\\
\dot{L_e^u} =&\ (D_1+\gamma D_2)S+\alpha_1 L_e^t-(\phi_1+\alpha+\mu)L_e^u\\
\dot{L_l^u} =&\ (1-p)\phi_1 L_e^u+\alpha_1 L_l^t-(\phi_2+\alpha+\mu)L_l^u\\
\dot{L_e^t} =&\ \alpha L_e^u+\alpha_3 L_e^p-(\alpha_1+\alpha_2+\mu)L_e^t\\
\dot{L_l^t} =&\ \alpha L_l^u-(\alpha_1+\alpha_2+\mu)L_l^t\\
\dot{J} =&\ \alpha_2(L_e^t+L_l^t)+r_2 R-[(D_1+\gamma D_2)+\mu]J\\
\dot{L_e^p} =&\ (D_1+\gamma D_2)(\xi_1 V+J)-(\phi_1+\alpha_3+\mu)L_e^p\\
\dot{L_l^p} =&\ (1-p)\phi_1 L_e^p-(\phi_2+\mu)L_l^p\\
\dot{D_e^u} =&\ p\phi_1(L_e^u+L_e^p)+\phi_2(L_l^u+L_l^p)+r_1 R-\textstyle\sum_{i=1}^6\rho_i D_e^u\\
\dot{D_i^u} =&\ \rho_i D_e^u-[(\psi_i+(1-\psi_i)(\tilde{\kappa}_i+\mu_d^u)]D_i^u \qquad i=1,\dots,6\\
\dot{D_i^d} =&\ \psi_i D_i^u+\tilde{\tau}_i T_i-[(\tau_i+(1-\tau_i)(\tilde{\kappa}_i+\mu_d^u)]D_i^d \qquad i=1,4\\
\dot{D_i^d} =&\ \psi_i D_i^u+\tilde{\tau}_i T_i+\hat{\tau}_i T_{i-1}-[(\tau_i+(1-\tau_i)(\tilde{\kappa}_i\mu_d^u)]D_i^d \quad i=2,3,5,6\\
\dot{T_i} =&\ \tau_i D_i^d-(\tilde{\tau}_i+\kappa_i+\mu_d)T_i \qquad i=3,6\\
\dot{T_i} =&\ \tau_i D_i^d-(\tilde{\tau}_i+\hat{\tau}_i+\kappa_i+\mu_d)T_i \qquad i=1,2,4,5\\
\dot{R} =&\ \textstyle\sum_{i=1}^6\kappa_i T_i+\sum_{i=1}^6\tilde{\kappa}_i D_i^u+\sum_{i=1}^6\tilde{\kappa}_i D_i^d-(r_1+r_2)R
\end{aligned} \tag{A}$$

where D is the 2×1 matrix $D=\bar{D^u}+\bar{D^d}$. In detail, $\bar{D^u}=\beta D^u\Delta$ with

$$D^u=\begin{vmatrix} D_1^u & D_2^u & D_3^u\\ D_4^u & D_5^u & D_6^u\end{vmatrix},$$

$$\Delta = \begin{vmatrix} 1 \\ \delta_1 \\ \delta_2 \end{vmatrix},$$

and $\bar{D^d} = \beta D^d \Delta$ with

$$D^d = \begin{vmatrix} D_1^d & D_2^d & D_3^d \\ D_4^d & D_5^d & D_6^d \end{vmatrix}.$$

Numbered indices correspond to the aforementioned six-fold split; $X_1 = X_{DS}^{SP}$, $X_2 = X_{MDR}^{SP}$, $X_3 = X_{XDR}^{SP}$, $X_4 = X_{DS}^{SN}$, $X_5 = X_{MDR}^{SN}$, and $X_6 = X_{XDR}^{SN}$. This applies only to D_i^u, D_i^d and T_i for $i = 1, \ldots, 6$. Parameter definitions can be found in Table A.

Parameter	Meaning	Value	Source
b	birth rate		calibration
μ	non-TB mortality rate		calibration
ν	vaccination rate		country data
β	transmission rate for SP DS		calibration
δ_1	MDR relative infectiousness (compared to DS)	1 (0.1-10)	[1]
δ_2	XDR relative infectiousness (compared to DS)	1 (0.1-10)	[1]
γ	SN infectiousness reduction (compared to SP)	0.22 (0.21-0.28)	[2, 3]
ξ_1	infectiousness reduction for vaccinated people	0.8 (0.73-0.81)	[4]
r_1	relapse rate	0.02	[5–7]
r_2	departure from treatment completion	1 (0.5-2.0)	[5–7]
ϕ_1	early latency progression rate	0.177 (0.01-0.25)	[8, 9]
	early latency progression rate in people living with HIV off ART	0.354 (0.02-0.5)	[8, 10]
ϕ_2	late latency progression rate to active TB	0.00185(0.00064-0.01)	[3, 8]
	late latency progression rate to active TB in people living with HIV off ART	0.0037 (0.00128-0.02)	[3, 10]
p	probability of active TB from early latency	0.2 (0.18-0.22)	[8]
	probability of active TB from early latency people living with HIV off ART	0.99	[8, 10]
α	rate of latency testing and treatment		country data
α_1	failed latency treatment		country data
α_2	rate of successfully treated latent cases		country data
α_3	rate of latent treatment of direct contacts of active cases		country data
ρ_1	rate of SP DS TB		calibration
ρ_2	rate of SP MDR TB		calibration
ρ_3	rate of SP XDR TB		calibration
ρ_4	rate of SN DS TB		calibration
ρ_5	rate of SN MDR TB		calibration
ρ_6	rate of SN XDR TB		calibration
ψ_i	testing rate for all 6 active TB compartments		country data
τ_i	treatment rate for all 6 active TB compartments		country data
$\tilde{\tau}_i$	treatment failure rate for all 6 active TB compartments		country data
$\hat{\tau}_i$	escalation of drug resistance following treatment	0	[11]
κ_i	recovery rate for all 6 active TB compartments		country data
$\tilde{\kappa}_i$	untreated recovery rate by smear (HIV-)	0.16 (0.03-0.16)	[12]
	untreated recovery rate by smear in people living with HIV off ART	0(0-0.03)	[12]
μ_d^u	all causes death rate (including disease) by smear in untreated individuals	0.12 (0.02-0.14)	[12]
	all causes death rate (including disease) by smear in people living with HIV	$\mu + \mu_d^u$	[12]
μ_d	disease related death rate by smear and strain		country data

Table A. Model parameters.

Vaccination

Vaccination against TB is generally part of the national childhood immunisation programme in most of the countries

where TB is endemic, reaching >80% of neonates and infants. Bacille Calmette-Guérin (BCG) vaccine has limited effect in preventing primary infection [4] and, if administered to already-infected individuals, does not prevent reactivation of latent pulmonary infection [13]. If administered to uninfected individuals, it can slow down the progression to active disease (following infection with the bacteria). Roy et al. [4] estimated limited protection against infection, with a protective efficacy of 19-27%, compared with 71% protective efficacy against reactivation to active TB, while protection by BCG against progression from infection to active disease, calculated by using infected individuals, was 58%.

LTBI progression

Results from a recent review [14] show the importance of distinguishing between people of varying disease-development rates by adding more than one latent compartment to TB models. Optima TB includes two compartments, early and late infection; all newly infected individuals enter the early latent stage and, if they do not develop active disease in the first 5 years after infection, move to the late latent compartment. This is in line with reports showing that 95% of active cases develop in the first 2 to 5 years after infection, with the other 5% over the rest of an individual's lifetime [15–17].

Parametrising the latent tuberculosis infection (LTBI) components of a model is one of the main issues in deterministic TB modelling, because countries do not collect these data and prevalence studies of latent infection are scarce. We initialise the LTBI compartments using estimates of annual trends [18]. We then calculate latency progression by applying a simple survival model with constant hazard, $CI = 1 - exp(-IR \times duration)$, to the number of infectious cases [8], where the cumulative incidence (CI) is a function of

incidence rate (IR) and time. These studies, however, are from the pre-chemotherapy era; these values are tentative starting points for calibration, to be adjusted when required.

LTBI treatment

Studies have shown that LTBI treatment is important towards the eradication of TB [19–22], because a quarter of the world population is estimated to be currently infected [18]. However, because of the widespread infection and the 10% lifetime probability of progressing to active TB, mass treatment would not be a cost-effective strategy. Moreover, even in high-burden circumscribed areas, mass screening and treatment have been proved to have no significant effect on tuberculosis due to the extremely high force of infection and loss of protection from infection once treatment is over [23]. For these reasons, WHO established a $\geq$ 90% preventive treatment target for 2025 only in high risk populations, such as people living with HIV or adult and child contacts of pulmonary TB cases [24]. In countries with high or upper-middle income and low TB burden (i.e. TB incidence of $<$100 per 100000 per year), WHO guidelines recommend testing and treatment of LTBI for people living with HIV, adult and child contacts of pulmonary TB cases, people initiating anti-tumour necrosis factor treatment, patients receiving dialysis, patients preparing for organ or haematological transplantation, and patients with silicosis. In prisoners, healthcare workers, immigrants from high TB burden countries, homeless persons and illicit drug users, systematic testing and treatment of LTBI is conditionally recommended, according to TB epidemiology and resource availability [25]. To test for LTBI, either commercial interferon-gamma release assays (IGRA) or Mantoux tuberculin skin testing (TST) could be used. Due to low TST specificity, BCG vaccination may result in false positive

results and an IGRA test would be required to give more precise results. Due to the higher costs of IGRA, however, TST is often the only test available. Before LTBI treatment, a chest radiography should be performed to rule out active TB disease. Recommended treatment regimens for LTBI include: 6 or 9 month isoniazid, 12 week rifapentine plus isoniazid, 3–4 month isoniazid plus rifampicin, or 3–4 month rifampicin alone [25].

In the model we include a secondary latency pathway collecting all individuals who have been previously treated for LTBI or TB and re-infected or infected after vaccination. This distinction is useful because these individuals will always result positive to TST testing and will not generally be treated for LTBI. The progression toward active TB from these compartment is reduced only in the 0-15 age group because of the effects of vaccination in children, while it is the same as the progression from the primary latent pathway for the older age groups because treatment does not entail immunisation.

Subclinical TB

The progression from LTBI to active TB can involve a state of asymptomatic disease [26–28]. Not much is known of this subclinical TB in terms of onset and duration. However, the undiagnosed individuals are potentially already infectious at this stage. Following subclinical TB, symptoms (which can include fever, weight loss and prolonged cough) start to appear and a diagnosis may follow. In the model D_e^u is a ‘junction’ compartment that merely collects and redistributes cases of newly activated TB across smears and strains; it does not contribute to the dynamics of the system. Thus,

$$\sum_{i=1}^{6} \rho_i D_e^u = p\phi_1 L_e^u + \phi_2 L_l^u + p\phi_1 L_e^p + \phi_2 L_l^p + r_1 R.$$

Smear status

The presence of acid-fast-bacilli (AFB) or other specimens on a sputum smear often indicates TB disease, and a posterior-anterior chest radiography is used to assess the possibility of pulmonary tuberculosis. Several studies have analysed and discussed specificity and sensitivity of different blood and/or sputum based TB tests [29–31]. smear-positive cases are generally assumed to be more infectious than smear-negative because of the higher amount of bacilli they expectorate [32]. The reduction of infectiousness in smear-negative cases with respect to smear-positive seems to be around 80%, however, studies analysing the number of secondary cases produced by primary active cases are either old and circumscribed to a delimited area [2, 33] or from the pre-chemotherapy era and unreproducible nowadays due to ethics.

Diagnosed vs undiagnosed TB

From a modelling perspective, this aspect of TB epidemiology is best supported by current data-availability. In conjunction with TB diagnosis, countries usually collect data on cases notified, drug-resistance status and co-infection with HIV. Data on smear status may also be available. Moreover, WHO publishes an annual TB report showing reports and estimates of incidence, diagnostic coverage, treatment uptake and deaths by geographic areas. The amount of time between disease onset and both diagnosis and treatment initiation, or, more specifically, the amount of time an individual stays infectious, combined with the rates of diagnosis and treatment uptake, is the main factor behind infection transmission. Styblo estimated that about 10 secondary infections arise annually from one untreated smear-positive case [32, 34]. Our model is informed by nationally collected

data on notified cases, treatment initiations and completion, loss to follow-up and number of deaths. Unsurprisingly, highest TB-related death rates usually occur in patients aged 65 and above because of more frequent drug-related adverse events and increased co-morbidity [35, 36]. Outputs from the model are calibrated against prevalence and incidence estimations annually calculated by WHO, which we disaggregate by age group according to local demographic rates. Similarly as for latency progression, problems arise in the calculation of natural recovery and death rates in the undiagnosed population, because they are inferred by using a simple survival model to very old datasets [12]. The resulting values should be considered as default values and calibrated, if needed, when analysing current epidemics.

Drug sensitivity

Once TB is diagnosed, one of the most significant pieces of information is the drug sensitivity of the strain. Multi-drug resistant (MDR) strains are resistant to the two main anti-TB drugs, isoniazid and rifampicin, while extensively drug resistant (XDR) strains are also resistant to any fluoroquinolone and at least one of three second-line injectable drugs (capreomycin, kanamycin and amikacin). Recent WHO global reports [37, 38] show that in 2016 there were 600,000 new cases of rifampicin-resistant (RR) TB globally, 490,000 of which were MDR-TB, challenging the prospect of ending TB by 2035. Treatment for drug-sensitive TB usually involves taking isoniazid and rifampicin for six months supplemented by ethambutol and pyrazinamide in the first two months [39], while treatment for drug resistant TB is longer and less efficacious and can last from 9 months to 2 years [37].

While it is possible for drug resistance to arise following improper treatment, it has been suggested that transmission

of drug-resistant strains has higher overall impact than primary escalation [40], i.e. mutations conferring resistance while on treatment. Moreover, very little information is currently available on strain escalation, countries do not usually collect this type of information. Thus, while the model includes strain escalation, the corresponding parameter default values are currently set to zero; they can be adjusted in case of data availability.

Recurrent TB

Finally, an important aspect of TB is its recurrence following treatment completion, but related data are not usually available. Several studies have analysed recurrence in TB [41–50], usually analysing small cohorts and reporting very different results, showing that numbers will vary according to several factors such as HIV status, drug resistance, and geography or, more precisely, different forces of infection in different areas. The WHO recording and reporting system distinguishes between new and re-treatment cases, however it does not discriminate between true relapse and reinfection, and it designates as relapse any recurrence of TB [51]. Since the majority of relapses arises during the first months up to 1-2 years after the conclusion of the first treatment [5–7], in order to differentiate between the two, we assume that cases of recurrence during the first 2 years following treatment completion are due to relapse, while later recurrences are due to reinfection. In the first case people move back to the undiagnosed TB compartments $\boldsymbol{D^u}$ according to a data-informed relapse-rate while, in the second case, move first to the successfully treated compartment $\boldsymbol{J}$ where they can proceed to the secondary latent pathway if re-infected.

Additional Results

Projections of active TB incidence (Figure A), prevalence (Figure B) and deaths (Figure C) to 2035.

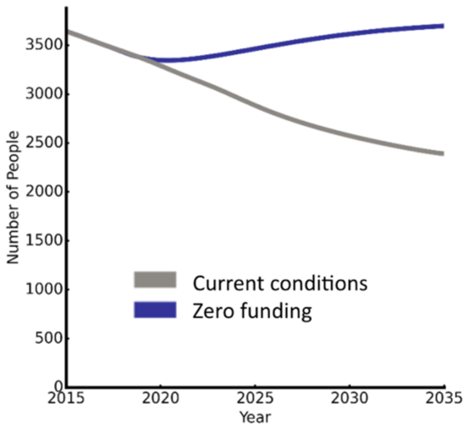

Fig A. New TB infections (all populations).

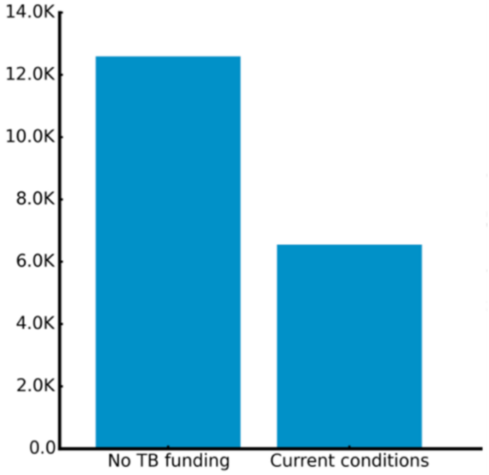

Fig B. Number of active TB infections (all populations).

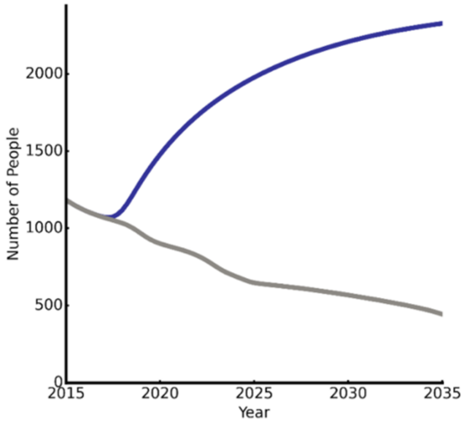

Fig C. Annual number of TB deaths (all populations).

References

1. Borrell S, Gagneux S. Infectiousness, reproductive fitness and evolution of drug-resistant Mycobacterium tuberculosis [State of the art]. The International Journal of Tuberculosis and Lung Disease. 2009;13(12):1456–1466.

2. Behr M, Warren S, Salamon H, Hopewell P, De Leon AP, Daley C, et al. Transmission of Mycobacterium tuberculosis from patients smear-negative for acid-fast bacilli. The Lancet. 1999;353(9151):444–449.

3. Abu-Raddad LJ, Sabatelli L, Achterberg JT, Sugimoto JD, Longini IM, Dye C, et al. Epidemiological benefits of more-effective tuberculosis vaccines, drugs, and diagnostics. Proceedings of the National Academy of Sciences. 2009;106(33):13980–13985.

4. Roy A, Eisenhut M, Harris RJ, Rodrigues LC, Sridhar S, Habermann S, et al. Effect of BCG vaccination against Mycobacterium tuberculosis infection in children: systematic review and meta-analysis. BMJ. 2014;349(aug04 5):g4643–g4643. doi:10.1136/bmj.g4643.

5. Driver CR, Munsiff SS, Li J, Kundamal N, Osahan SS. Relapse in persons treated for drug-susceptible tuberculosis in a population with high coinfection with human immunodeficiency virus in New York City. Clinical infectious diseases. 2001;33(10):1762–1769.
6. Becerra MC, Appleton SC, Franke MF, Chalco K, Bayona J, Murray MB, et al. Recurrence after treatment for pulmonary multidrug-resistant tuberculosis. Clinical infectious diseases. 2010;51(6):709–711.
7. Jo KW, Yoo JW, Hong Y, Lee JS, Lee SD, Kim WS, et al. Risk factors for 1-year relapse of pulmonary tuberculosis treated with a 6-month daily regimen. Respiratory medicine. 2014;108(4):654–659.
8. Andrews JR, Noubary F, Walensky RP, Cerda R, Losina E, Horsburgh CR. Risk of progression to active tuberculosis following reinfection with Mycobacterium tuberculosis. Clinical infectious diseases. 2012;54(6):784–791.
9. Menzies NA, Cohen T, Lin HH, Murray M, Salomon JA. Population health impact and cost-effectiveness of tuberculosis diagnosis with Xpert MTB/RIF: a dynamic simulation and economic evaluation. PLoS medicine. 2012;9(11).
10. Sonnenberg P, Glynn JR, Fielding K, Murray J, Godfrey-Faussett P, Shearer S. How soon after infection with HIV does the risk of tuberculosis start to increase? A retrospective cohort study in South African gold miners. Journal of Infectious Diseases. 2005;191(2):150–158.
11. Cohen T, Murray M. Modeling epidemics of multidrug-resistant M. tuberculosis of heterogeneous fitness. Nature medicine. 2004;10(10):1117–1121.

12. Tiemersma EW, van der Werf MJ, Borgdorff MW, Williams BG, Nagelkerke NJ. Natural history of tuberculosis: duration and fatality of untreated pulmonary tuberculosis in HIV negative patients: a systematic review. PloS one. 2011;6(4).
13. Abubakar I, Pimpin L, Ariti C, Beynon R, Mangtani P, Sterne J, et al. Systematic review and meta-analysis of the current evidence on the duration of protection by bacillus Calmette–Guérin vaccination against tuberculosis. Health Technol Assess. 2013;17(37). doi:10.3310/hta17370.
14. Menzies NA, Wolf E, Connors D, Bellerose M, Sbarra AN, Cohen T, et al. Progression from latent infection to active disease in dynamic tuberculosis transmission models: a systematic review of the validity of modelling assumptions. The Lancet Infectious Diseases. 2018;18(8):e228–e238. doi:10.1016/s1473-3099(18)30134-8.
15. Styblo K. The relationship between the risk of tuberculous infection and the risk of developing infectious tuberculosis. Bull IUAT. 1985;60(3):117–119.
16. Dye C. Breaking a law: tuberculosis disobeys Styblo's rule; 2008.
17. Aparicio JP, Castillo-Chavez C. Mathematical modelling of tuberculosis epidemics. Math Biosci Eng. 2009;6(2):209–37.
18. Houben RM, Dodd PJ. The global burden of latent tuberculosis infection: a re-estimation using mathematical modelling. PLoS medicine. 2016;13(10):e1002152.
19. Ziv E, Daley CL, Blower SM. Early therapy for latent tuberculosis infection. American journal of epidemiology. 2001;153(4):381–385.

20. Sterling TR, Bethel J, Goldberg S, Weinfurter P, Yun L, Horsburgh CR, et al. The scope and impact of treatment of latent tuberculosis infection in the United States and Canada. American journal of respiratory and critical care medicine. 2006;173(8):927–931.

21. Cohn DL. Treatment of latent tuberculosis infection: renewed opportunity for tuberculosis control. Clinical infectious diseases. 2000;31(1):120–124.

22. Young DB, Gideon HP, Wilkinson RJ. Eliminating latent tuberculosis. Trends in microbiology. 2009;17(5):183–188.

23. Churchyard GJ, Fielding KL, Lewis JJ, Coetzee L, Corbett EL, Godfrey-Faussett P, et al. A trial of mass isoniazid preventive therapy for tuberculosis control. New England Journal of Medicine. 2014;370(4):301–310.

24. World Health Organization. Gear up to end TB: introducing the end TB strategy. World Health Organization; 2015.

25. Getahun H, Matteelli A, Abubakar I, Aziz MA, Baddeley A, Barreira D, et al. Management of latent Mycobacterium tuberculosis infection: WHO guidelines for low tuberculosis burden countries. European Respiratory Journal. 2015;46(6):1563–1576. doi:10.1183/13993003.01245-2015.

26. Heemskerk D, Caws M, Marais B, Farrar J. Tuberculosis in Adults and Children. Springer; 2015.

27. Achkar JM, Jenny-Avital ER. Incipient and subclinical tuberculosis: defining early disease states in the context of host immune response. Journal of Infectious Diseases. 2011;204(suppl_4):S1179–S1186.

28. Pai M, Behr MA, Dowdy D, Dheda K, Divangahi M, Boehme CC, et al. Tuberculosis. Nature Reviews Disease Primers. 2016;2(1). doi:10.1038/nrdp.2016.76.

29. Nyendak MR, Lewinsohn DA, Lewinsohn DM. New diagnostic methods for tuberculosis. Current opinion in infectious diseases. 2009;22(2):174.

30. Boehme CC, Nicol MP, Nabeta P, Michael JS, Gotuzzo E, Tahirli R, et al. Feasibility, diagnostic accuracy, and effectiveness of decentralised use of the Xpert MTB/RIF test for diagnosis of tuberculosis and multidrug resistance: a multicentre implementation study. The Lancet. 2011;377(9776):1495–1505. doi:10.1016/s0140-6736(11)60438-8.

31. Lyra JMAd, Maruza M, Verza M, Carneiro MM, Albuquerque MdFMd, Rossetti ML, et al. Evaluation of four molecular methods for the diagnosis of tuberculosis in pulmonary and blood samples from immunocompromised patients. Memórias do Instituto Oswaldo Cruz. 2014;109(6):805–813.

32. Sepkowitz KA. How contagious is tuberculosis? Clinical Infectious Diseases. 1996;23(5):954–962.

33. Hernandez-Garduno E, Cook V, Kunimoto D, Elwood R, Black W, FitzGerald J. Transmission of tuberculosis from smear negative patients: a molecular epidemiology study. Thorax. 2004;59(4):286–290.

34. Styblo K. Etat actuel de la question. I. Epidemiologie de la tuberculose. Bull Int Union Tuberc. 1978;53(3):153–166.

35. Liaw YS, Yang PC, Yu CJ, Wu ZG, Chang DB, Lee LN, et al. Clinical spectrum of tuberculosis in older patients. Journal of the American Geriatrics Society. 1995;43(3):256–260.

36. Negin J, Abimbola S, Marais BJ. Tuberculosis among older adults–time to take notice. International Journal of Infectious Diseases. 2015;32:135–137.

37. Falzon D, Schünemann HJ, Harausz E, González-Angulo L, Lienhardt C, Jaramillo E, et al. World Health Organization treatment guidelines for drug-resistant tuberculosis, 2016 update. European Respiratory Journal. 2017;49(3).

38. World Health Organization. Global Tuberculosis Report 2017. World Health Organization; 2017. Available from: https://www.ebook.de/de/product/31660606/world_health_organization_global_tuberculosis_report_2017.html.

39. World Health Organization. Guidelines for Treatment of Drug-Susceptible Tuberculosis and Patient Care: 2017 Update. World Health Organization; 2017. Available from: https://www.ebook.de/de/product/29978796/world_health_organization_guidelines_for_treatment_of_drug_susceptible_tuberculosis_and_patient_care_2017_update.html.

40. McBryde ES, Meehan MT, Doan TN, Ragonnet R, Marais BJ, Guernier V, et al. The risk of global epidemic replacement with drug-resistant Mycobacterium tuberculosis strains. International Journal of Infectious Diseases. 2017;56:14–20.

41. Moodley Y, Govender K. A systematic review of published literature describing factors associated with tuberculosis recurrence in people living with HIV in Africa. African health sciences. 2015;15(4):1239–1246.

42. Wang JY, Lee LN, Lai HC, Hsu HL, Liaw YS, Hsueh PR, et al. Prediction of the tuberculosis reinfection

proportion from the local incidence. The Journal of infectious diseases. 2007;196(2):281–288.

43. Hatherell HA, Colijn C, Stagg HR, Jackson C, Winter JR, Abubakar I. Interpreting whole genome sequencing for investigating tuberculosis transmission: a systematic review. BMC medicine. 2016;14(1):21.

44. Lambert ML, Hasker E, Van Deun A, Roberfroid D, Boelaert M, Van der Stuyft P. Recurrence in tuberculosis: relapse or reinfection? The Lancet infectious diseases. 2003;3(5):282–287.

45. Korenromp EL, Scano F, Williams BG, Dye C, Nunn P. Effects of human immunodeficiency virus infection on recurrence of tuberculosis after rifampin-based treatment: an analytical review. Clinical Infectious Diseases. 2003;37(1):101–112.

46. Sonnenberg P, Murray J, Glynn JR, Shearer S, Kambashi B, Godfrey-Faussett P. HIV-1 and recurrence, relapse, and reinfection of tuberculosis after cure: a cohort study in South African mineworkers. The Lancet. 2001;358(9294):1687–1693.

47. Glynn JR, Murray J, Bester A, Nelson G, Shearer S, Sonnenberg P. High rates of recurrence in HIV-infected and HIV-uninfected patients with tuberculosis. The Journal of infectious diseases. 2010;201(5):704–711.

48. Cox H, Kebede Y, Allamuratova S, Ismailov G, Davletmuratova Z, Byrnes G, et al. Tuberculosis Recurrence and Mortality after Successful Treatment: Impact of Drug Resistance. PLoS Medicine. 2006;3(10):e384. doi:10.1371/journal.pmed.0030384.

49. Migliori G, Espinal M, Danilova I, Punga V, Grzemska M, Raviglione M. Frequency of recurrence among MDR-TB cases 'successfully' treated with standardised

short-course chemotherapy. International Journal of Tuberculosis and Lung Disease. 2002;6(10):858–864.

50. Blower SM, Mclean AR, Porco TC, Small PM, Hopewell PC, Sanchez MA, et al. The intrinsic transmission dynamics of tuberculosis epidemics. Nature medicine. 1995;1(8):815–821.

51. Zignol M, Wright A, Jaramillo E, Nunn P, Raviglione MC. Patients with previously treated tuberculosis no longer neglected. Clinical infectious diseases. 2007;44(1):61–64.

Legend

Transmission model and epidemiological rationale.

Model's equations (Eq A);

Table of parameters (Table A);

Projections of new TB infections to 2035 (S1 Fig A);

Number of active TB infections with and without funding to current TB programs (Fig B);

Projections of yearly TB deaths to 2035 (Fig C).
