# Supplementary material for: Optima TB: A tool to help optimally allocate tuberculosis spending
Source: PLoS Comput Biol. 2021 Sep 27;17(9):e1009255. doi: 10.1371/journal.pcbi.1009255 (PMC8496838; doi:10.1371/journal.pcbi.1009255)
Supplement: S2 File — (Table A in S2 File) TB treatment and care cost assumptions; (Table B in S2 File) TB treatment intervention effectiveness inputs; and (Table C in S2 File) TB screening and diagnosis intervention inputs for yield. (DOC) [file pcbi.1009255.s002.doc]

**Supplement 2: Intervention cost and effect inputs**

The cost inputs for this analysis were derived from a range of in-country sources. These sources included in-country procurement records, Tuberculosis (TB) programme reviews, WHO national health accounts for TB, expert opinion and specific calculations performed for the purpose of this analysis. The cost of TB treatment in Belarus constitutes the largest proportion of total TB expenditure in Belarus. A detailed expenditure and cost-analysis was therefore carried out to estimate the cost of different existing and prospective treatment modalities.

Table 1 summarises how treatment costs by TB drug-resistance type and modality were estimated using a per diem approach. The calculation was based on the treatment duration for each of the different modalities and the unit costs per treatment day inside and outside of hospital settings. The latter were established based on the total TB treatment and care expenditure (excluding drug costs) and the number of days of patients in in-patient and outpatient care. On that basis, the cost of care per person for a full course of treatment (excluding drug costs) was established for the different TB drug-resistance types and delivery modalities. Other costs including incentives and food packages were considered for the relevant modalities. Drug costs for the different TB types were added based on information from country and Global Fund procurement processes. Based on all of these sources, the cost of a full course of treatment was estimated for 15 different treatment combinations. For treatment types with durations beyond one calendar year, the annualised cost (cost incurred within a calendar year) was calculated.

**Table A: TB treatment and care cost assumptions***

| **Treatment modality** | **Treatment regimen group** | **Duration (in days)** | | | **Unit costs per day** | | **Costs of care** | **Other costs** | | | **Total non-drug costs** | | **Drug costs** | **Total costs** | | |
| --- | --- | --- | --- | --- | --- | --- | --- | --- | --- | --- | --- | --- | --- | --- | --- | --- |
|  |  | **Inpatient** | **Ambulatory** | **Total** | **Inpatient** | **Ambulatory** | **Total** | **Food packages** | | **Incentives** | |  | **Full course** | | **Full course** | **Annualised** |
| Hospital-focussed | DS-TB | 60 | 120 | 180 | 24.80 | 8.36 | 2,492 | 63 | - | | 2,555 | | 55 | **2,610** | | 2,610 |
| Hospital-focussed | MDR-TB | 210 | 390 | 600 | 32.90 | 8.36 | 10,170 | 206 | - | | 10,376 | | 3,782 | **14,158** | | 8,613 |
| Hospital-focussed | XDR-TB | 270 | 450 | 720 | 32.90 | 8.36 | 12,646 | 237 | - | | 12,883 | | 7,600 | **20,483** | | 10,384 |
| Standard ambulatory | DS-TB | 14 | 166 | 180 | 24.80 | 8.36 | 1,735 | 88 | - | | 1,823 | | 55 | **1,878** | | 1,878 |
| Standard ambulatory | MDR-TB | 45 | 555 | 600 | 32.90 | 8.36 | 6,122 | 292 | - | | 6,414 | | 3,782 | **10,196** | | 6,203 |
| Standard ambulatory | MDR-TB short-course | 30 | 285 | 315 | 32.90 | 8.36 | 3,370 | 150 | - | | 3,520 | | 1,000 | **4,520** | | 4,520 |
| Standard ambulatory | XDR-TB | 60 | 660 | 720 | 32.90 | 8.36 | 7,493 | 348 | - | | 7,841 | | 7,600 | **15,441** | | 7,828 |
| Incentivised ambulatory | DS-TB | 14 | 166 | 180 | 24.80 | 8.36 | 1,735 | 87 | 338 | | 2,160 | | 55 | **2,215** | | 2,215 |
| Incentivised ambulatory | MDR-TB | 45 | 555 | 600 | 32.90 | 8.36 | 6,122 | 292 | 1,129 | | 7,543 | | 3,782 | **11,325** | | 6,889 |
| Incentivised ambulatory | MDR-TB short-course | 30 | 285 | 315 | 32.90 | 8.36 | 3,370 | 150 | 580 | | 4,100 | | 1,000 | **5,100** | | 5,100 |
| Incentivised ambulatory | MDR-TB new drugs^1^ | 45 | 555 | 600 | 32.90 | 8.36 | 6,122 | 292 | 1,129 | | 7,543 | | 7,253 | **14,796** | | 9,001 |
| Incentivised ambulatory | XDR-TB | 60 | 660 | 720 | 32.90 | 8.36 | 7,493 | 348 | 1,342 | | 9,183 | | 7,600 | **16,783** | | 8,508 |
| Incentivised ambulatory | XDR-TB new drugs^1^ | 60 | 660 | 720 | 32.90 | 8.36 | 7,493 | 348 | 1,342 | | 9,183 | | 13,853 | **23,036** | | 11,678 |
| Involuntary isolation | MDR-TB | 600 | - | 600 | 29.50 | - | 17,700 | - | - | | 17,700 | | 3,782 | **45,588^2^** | | 27,353 |
| Involuntary isolation | XDR-TB | 720 | - | 720 | 29.50 | - | 21,240 | - | - | | 21,240 | | 7,600 | **45,588^2^** | | 27,353 |

^*^ All values presented are in 2015 USD, $. All unit costs include the cost of service delivery in addition to medicine, test kits, etc. All treatment unit costs are per DS-TB, MDR-TB or XDR-TB case.

^1^ New drug regimens include Bedaquiline and Linezolid in addition to the standard background regimen until smear conversion.

^2^ The annual unit cost of $45,588 was estimated using a top-down approach. Reported national spending on involuntary isolation for people living with MDR-TB or XDR-TB was divided by the estimated number of people receiving treatment in such facilities. Using a bottom-up approach generated unit costs of $21,482 and $28,840 for people receiving MDR-TB or XDR-TB treatment respectively. The difference between the two estimates can be explained by the high security costs associated with the intervention as patients are monitored by security personnel throughout their stay. Unit costs estimated using the bottom-up approach capture only clinical costs and the top-down estimates were therefore used in the analysis.

**Table B: TB treatment intervention effectiveness inputs**

| **Intervention** | **Effectiveness** | **Adherence** | **Source** |
| --- | --- | --- | --- |
| Hospital Focussed DS-TB | 85% | 93% | [1] |
| Hospital Focussed MDR-TB | 49% | 61% | [1] |
| Hospital Focussed XDR-TB | 38% | 40% | [2],[1] outcomes from Homel Oblast |
| Ambulatory DS-TB | 85% | 93% | Assumed no change in effectiveness or adherence compared to hospital focussed interventions. Studies such as [3] have shown that ambulatory care does not reduced the quality of treatment compared with extended hospitalisation. |
| Ambulatory MDR-TB | 49% | 61% |
| Ambulatory MDR-TB Short-Course | 64% | 78% | [4] |
| Ambulatory XDR-TB | 38% | 40% | Same assumption made as for ambulatory DS-TB and MDR-TB treatment |
| Incentivised Ambulatory DS-TB | 85% | 95% | Increase in percentage of adherence based on [5]. |
| Incentivised Ambulatory MDR-TB | 49% | 71% |
| Incentivised Ambulatory MDR-TB Short Course | 64% | 84% |
| Incentivised Ambulatory XDR-TB | 38% | 56% |
| Incentivised Ambulatory New Drugs MDR-TB | 92% | 71% |
| Incentivised Ambulatory New Drugs XDR-TB | 92% | 56% |
| Involuntary Isolation MDR-TB | 49% | 100% | Assumed no change in effectiveness compared to hospital focussed interventions given that the same treatment regimens are provided, but adherence was assumed to be 100% based on expert opinion and security to guard the buildings. |
| Involuntary Isolation XDR-TB | 38% | 100% |
| IPT for General Population | 39% | 50% | [6],[7],[8] |
| IPT for PLHIV | 33% | 82% | [9],[10] |
| BCG Vaccination (0-4yr olds) | 50% | - | [11],[12] |

**Table C: TB screening and diagnosis intervention inputs for yield**

| **Screening strategies** | **Yield** | **Source** |
| --- | --- | --- |
| Mass Screening with X-ray (General Population, Obligatory Groups) | 0.025% | Yield of mass screening, value reported for Minsk (year 2011), where 187 active TB infections were identified out of 733,414 screens. de [13] |
| Active Case Finding (populations with high-exposure to TB: PLHIV, homeless, people who inject drugs, etc.) | 0.029% | Average lower-bound yield of ACF in low-incidence setting for relevant populations. [14] |
| Incentivised Active Case Finding | 0.033% | Average higher-bound yield of ACF in low-incidence setting for relevant populations. [14] |
| Contact Tracing | 0.190% | Most recent reported yield (year 2014). de [15] |
| Incentivised Contact Tracing | 0.470% | Assumed highest yield achieved historically (year 2013) in [16]. |

Population groups and prospective interventions were defined in consultation with local stakeholders, which included: the World Bank, the Republican Scientific and Practice Centre for Pulmonology and Tuberculosis, the Republican Scientific and Practice Centre for Medical Technologies, the World Health Organization, the Global Fund to Fight AIDS, Tuberculosis and Malaria, and the Ministry of Public Health of the Republic of Belarus. Demographic, epidemiological, programmatic, cost and spending data for the populations and interventions defined were collated in partnership with stakeholders. Stakeholders informed and validated assumptions for the analysis.

**References**

1. Gurbanova E. GLC/Europe Mission for Monitoring of the Implementation of the National M/XDR-TB Response Plan. Minsk: World Health Organization; 2017.
2. World Health Organisation. Belarus: Tuberculosis Profile. World Health Organisation. 2016
3. Bassili A, Fitzpatrick C, Qadeer E, Fatima R, Floyd K, Jaramillo E. A systematic review of the effectiveness of hospital-and ambulatory-based management of multidrug-resistant tuberculosis. The American journal of tropical medicine and hygiene. 2013 Aug 7;89(2):271-80.
4. Kibret KT, Moges Y, Memiah P, Biadgilign S. Treatment outcomes for multidrug-resistant tuberculosis under DOTS-Plus: a systematic review and meta-analysis of published studies. Infectious diseases of poverty. 2017 Dec 1;6(1):7.
5. Nguyen, L. Progress on updating WHO guidance on support to treatment adherence and model of care. Workshop: Innovative WHO policies to support the End TB Strategy, Union Conference. Liverpool, 26 October 2016.
6. Menzies D, Al Jahdali H, Al Otaibi B. Recent developments in treatment of latent tuberculosis infection. The Indian journal of medical research. 2011 Mar;133(3):257.
7. Stagg HR, Zenner D, Harris RJ, Munoz L, Lipman MC, Abubakar I. Treatment of latent tuberculosis infection: a network meta-analysis. Annals of internal medicine. 2014 Sep 16;161(6):419-28.
8. Getahun H, Matteelli A, Abubakar I, Aziz MA, Baddeley A, Barreira D, Den Boon S, Gutierrez SM, Bruchfeld J, Burhan E, Cavalcante S. Management of latent Mycobacterium tuberculosis infection: WHO guidelines for low tuberculosis burden countries. European Respiratory Journal. 2015 Dec 1;46(6):1563-76.
9. Ayele HT, van Mourik MS, Debray TP, Bonten MJ. Isoniazid prophylactic therapy for the prevention of tuberculosis in HIV infected adults: a systematic review and meta-analysis of randomized trials. PLoS One. 2015;10(11).
10. World Health Organization. Guidelines on the management of latent tuberculosis infection. World Health Organization; 2015 Jan 15.
11. Roy A, Eisenhut M, Harris RJ, Rodrigues LC, Sridhar S, Habermann S, et al. Effect of BCG vaccination against Mycobacterium tuberculosis infection in children: systematic review and meta-analysis. BMJ. 2014;349(aug04 5):g4643{g4643.doi:10.1136/bmj.g4643.
12. Pai M, Behr MA, Dowdy D, Dheda K, Divangahi M, Boehme CC, et al. Tuberculosis. Nature Reviews Disease Primers. 2016;2(1). doi:10.1038/nrdp.2016.76.
13. de Colombani P. Review of the national tuberculosis programme in Belarus, 10-21 October 2011. World Health Organization; 2012.
14. Shapiro AE, Chakravorty R, Akande T, Lonnroth K, Golub JE. A systematic review of the number needed to screen to detect a case of active tuberculosis in different risk groups. Geneva: World Health Organization; 2013.
15. de Colombani P, Veen J. Review of the national tuberculosis programme in Belarus, 8-18 December 2015. World Health Organization; 2016.
16. Hovhannesyan A, Dadu A, Astrauko A, Skrahina A. TB Epidemiological and Impact Analysis in Belarus. Copenhagen: WHO Regional Office for Europe; 2015.

**Legend**

Intervention cost and effect inputs.
TB treatment and care cost assumptions (Table A);
TB treatment intervention effectiveness inputs (Table B);
TB screening and diagnosis intervention inputs for yield (Table C).
