# Supplementary material for: Optima TB: A tool to help optimally allocate tuberculosis spending
Source: PLoS Comput Biol. 2021 Sep 27;17(9):e1009255. doi: 10.1371/journal.pcbi.1009255 (PMC8496838; doi:10.1371/journal.pcbi.1009255)
Supplement: S3 File — (DOCX) [file pcbi.1009255.s003.docx]

**Summary of modelling process in relation to TB MAC country level modelling guidance**

**1. Relevance: Modelling should assess relevant policies and outcomes**

Before starting the application, a scope of work was defined with local stakeholders to guide the model application. In consultation with the modelling team, local stakeholders and decision makers identified policy questions, scenarios, relevant optimisation outcomes and constraints for the application.

**2. Realism: Modelling should consider implementation challenges and examine requirements for policy success**

Where data was unavailable, assumptions were informed or reviewed by the local experts involved in policy and programme implementation. All data sources and assumptions were documented during the application process. Constraints were also defined by local experts and stakeholders to reflect logistic, ethical, political, and financial barriers for scaling-up or defunding specific interventions.

**3. Appropriateness of model structure: Model design should be justified in terms of the policy questions being considered and avoid unnecessary complexity**

Optima TB was applied to answer the questions defined in the scope of work based on available data. The model was able to capture the main NTP interventions for TB care and control as well as prospective interventions under consideration to answer the scope of work questions by drawing on routine and country data as much as possible.

**4. Consideration of all evidence: Modelling should consider all available evidence relevant to the decision problem**

A rapid review of relevant regional and country-level peer-reviewed and grey literature was carried out during the model application. Where local data was unavailable, evidence was appraised and local experts advised on the appropriateness of evidence to the Belarussian context. Different data were triangulated to identify and address any potential conflicting evidence (e.g. number of routinely reported notifications vs. annual number of tests through different modalities and their respective yields). Where local data were available, these were reviewed with local experts to evaluate appropriateness and local experts were consulted and reviewed each stage of the application (demographic fitting, epidemiological calibration, costing and estimated impact of existing spending, scenario analyses and optimisation results).

**5. Validation: Results should be compared to evidence not used for model parameterization or calibration**

Input data were validated through appraisal with local experts, triangulation where possible and checked against routinely reported data (e.g. notifications, which are not input data and are instead calibrated against) as well as key TB estimates (e.g. incidence/prevalence, which only help initialise the model). Local experts were involved throughout the model application and reviewed each stage of the application informing iterations within each stage (demographic fitting, epidemiological calibration, costing and estimated impact of existing spending, scenario analyses and optimisation results). Local stakeholders were consulted for results at key stages during model application (calibration/costing and scenarios, optimisation).

**6. Informativeness: Modelling should report results for a wide range of outcomes**

Key indicators identified during the scope of work guided the main model outputs and optimisation objectives (mortality reduction, incidence reduction and prevalence reduction). Various time horizons were investigated and the impact of various scenarios of interest to local experts and stakeholders as well as the optimisation outputs reported consequences on progress toward key national policy targets and global End TB milestones and targets.

**7. Transparency: Reporting should include a description of supporting evidence, limitations, sensitivity analyses and conflicts of interest**

The model structure and application methodology were explained in detail to country experts and key stakeholders. Both model structure and application were published in a report shortly after the analysis was completed: <https://openknowledge.worldbank.org/bitstream/handle/10986/27475/116896.pdf?sequence=5&isAllowed=y>. The report, along with slides and presentations to local experts and stakeholders described baseline and policy scenarios in detail. Slides and presentations to local experts and stakeholders conveyed data sources, validation and limitations using non-technical language. Key stakeholders in the country were involved and consulted to review outputs of key stages during the application process (calibration/costing and scenarios, optimisation).

**8. Timeliness: Modelling should provide results in time for decisions to be made**

Deadlines for analysis outputs were set out at the scope of work stage so that outputs could be reviewed and revised as required in time for key policy meetings. As mentioned in the discussion section of the paper: “The timeliness of the Belarus analysis helped inform dialogue on national TB care, including a round-table consultation organised by the WHO country office in Minsk in 2017 and a regional reform meeting in Bishkek, during which cornerstones of reform were agreed. This analysis has informed ongoing activities for TB financing and planning by the WHO Regional Office for Europe and Ministry of Health, and the findings have helped advocate for more and better quality ambulatory care.”

**9. Country ownership: Modelling should be conducted through participation with local stakeholder**

Local stakeholders, in the context of their collaboration with the Global Fund on AIDS, TB and Malaria, requested technical assistance from the World Bank who in collaboration with local stakeholders selected Optima TB for application. Local experts and key stakeholders were engaged extensively throughout the modelling application. Local experts contributed throughout and key stakeholders reviewed and provided input to inform iterations at key stages of the analysis as mentioned above (calibration/costing and scenarios, optimisation).

**10. Iteration: Modelling should be an iterative process, and reconsidered given new evidence**

As mentioned above, local stakeholders reviewed outputs at key stages that in turn informed iterations of the model application and outputs. The need to repeat analyses when better data becomes available, in line with strategic plan timelines (between every 3-5 years), or when notable new policy decisions are being considered was clearly stated and conveyed to local experts and stakeholders.
